# Global burden of *Clostridium difficile* infections: a systematic review and meta-analysis

**DOI:** 10.7189/jogh.09.010407

**Published:** 2019-06

**Authors:** Evelyn Balsells, Ting Shi, Callum Leese, Iona Lyell, John Burrows, Camilla Wiuff, Harry Campbell, Moe H Kyaw, Harish Nair

**Affiliations:** 1 Centre for Global Health Research, Usher Institute of Population Health Sciences and Informatics, University of Edinburgh, Edinburgh, UK; 2 Health Protection Scotland, Glasgow, UK; 3 Sanofi Pasteur, Swiftwater, Pennsylvania, USA; * Joint first authorship; ** Joint last authorship

## Abstract

**Background:**

*Clostridium difficile* is a leading cause of morbidity and mortality in several countries. However, there are limited evidence characterizing its role as a global public health problem. We conducted a systematic review to provide a comprehensive overview of *C. difficile* infections (CDI) rates.

**Methods:**

Seven databases were searched (January 2016) to identify studies and surveillance reports published between 2005 and 2015 reporting CDI incidence rates. CDI incidence rates for health care facility-associated (HCF), hospital onset-health care facility-associated, medical or general intensive care unit (ICU), internal medicine (IM), long-term care facility (LTCF), and community-associated (CA) were extracted and standardized. Meta-analysis was conducted using a random effects model.

**Results:**

229 publications, with data from 41 countries, were included. The overall rate of HCF-CDI was 2.24 (95% confidence interval CI = 1.66-3.03) per 1000 admissions/y and 3.54 (95%CI = 3.19-3.92) per 10 000 patient-days/y. Estimated rates for CDI with onset in ICU or IM wards were 11.08 (95%CI = 7.19-17.08) and 10.80 (95%CI = 3.15-37.06) per 1000 admission/y, respectively. Rates for CA-CDI were lower: 0.55 (95%CI = 0.13-2.37) per 1000 admissions/y. CDI rates were generally higher in North America and among the elderly but similar rates were identified in other regions and age groups.

**Conclusions:**

Our review highlights the widespread burden of disease of *C. difficile*, evidence gaps, and the need for sustainable surveillance of CDI in the health care setting and the community.

*Clostridium difficile* is a leading cause of health care-associated infections (HAIs) and an important public health threat. *C. difficile* has been associated with substantial morbidity and mortality worldwide and among individuals of all ages beyond the traditionally recognized at-risk groups (eg, elderly, hospitalized patients, or those under antimicrobial therapy) [1]. In the United States, *C. difficile* caused an estimated half a million infections and 29 000 deaths in 2012 [2]. Almost two thirds of these were associated with inpatient care, and more than 80% of these deaths occurred in those 65 years and over [2]. Challenges remain to control incidence of *C. difficile* infections (CDI) rates in other world regions such as Europe [3-5] and Asia [6]. It is estimated that approximately 40 000 cases among inpatients are potentially underdiagnosed each year in Europe [5]. Furthermore, recurrence of CDI is estimated to occur among a considerable percentage of cases (approximately 20%-30%) [7]. Though the global health care costs associated with CDI are not known, these are likely to be substantial with estimates suggesting attributable costs of US$ 5.4-6.3 billion per year in the United States [8].

Considering the evolving epidemiology of *C. difficile* morbidity and mortality, global challenges regarding antibiotic stewardship and limited alternative preventative options for CDI, it is important to assess its burden to inform public health action. The emergence of hyper virulent strain PCR ribotype 027/NAP1, significant increases in incidence of hospitalizations associated with *C. difficile* by the mid-2000s, and outbreaks of CDI in hospitals globally are examples of the major impact *C. difficile* can have on health care systems [1,9-12]. Through the implementation of standardized surveillance case definitions, a considerable proportion of cases of CDI occurring outside hospitals has also been identified [1,13-15]. Increased awareness of the role of *C. difficile* as a global health problem is required to reduce morbidity and control rates of CDI [16,17]. However, there are limited evidence characterizing its role as a global public health problem. We aimed to conduct a comprehensive examination of globally reported rates of CDI incidence, in order to develop baseline epidemiological estimates of incidence and identify characteristics and gaps in the available evidence base.

## METHODS

### Identification of eligible publications

We searched seven electronic databases (Medline, Embase, Global Health (through Ovid), CINAHL, LILACS, WHO Library, and Web of Science) in January 2016 to identify publications reporting incidence rates of CDI. Search strategies were developed with the assistance of a medical librarian and included a combination of MeSH and keywords relevant for CDI and incidence or burden of disease. An internet search to identify the most recent data from national surveillance reports was also conducted to supplement evidence from published literature (Table S1 in **Online Supplementary Document**). Details on eligibility criteria for the review are described in **Table 1**. Two reviewers (EB, TS) screened the records obtained through electronic medical databases and identified articles that met the inclusion criteria. We included studies and surveillance reports with a publication year between 2005 and 2015 inclusive and reporting CDI cases identified through positive laboratory assay or endoscopic findings using surveillance case definitions [18] or administratively coded CDI hospitalizations (eg, International Classification of Diseases (ICD) ICD-9-ICM = 00845.0 or ICD-10 = A04.7). Studies focusing on recurrence, asymptomatic colonization or mortality, or those published in languages other than English or Spanish were excluded. We also excluded data from periods of outbreaks or from intervention arms of infection control studies.

**Table 1:** Eligibility criteria

Inclusion criteria
Observational (during non-outbreak periods) or interventional studies (pre-intervention period) and national surveillance reports published between 2005 and 2015.
English and Spanish language (for peer-reviewed publications).
Publication reports rates of CDI incidence for individuals of all ages in any of the following settings: hospitals, medical intensive care units, internal medicine wards, long term care facilities or nursing homes and in the community.
Case ascertainment methods and case definition compatible with current CDI surveillance (laboratory or histological-diagnosis) or administrative coded hospitalizations (ICD9-9-CM:008.45; ICD-10:A04.7) are clearly reported.
Data on incidence of CDI rate can be extracted as an independent outcome and at least two of the following are available: number of cases during study period, study population (denominator) or rate.
Exclusion criteria:
Articles published before 2005 or reporting data for a study population already considered in the review.
Methods for case ascertainment are not clearly reported.
Interventional before-and-after studies without clear background rate or studies reporting rates outbreak periods exclusively.
C. difficile assessed as a co-infection, composite outcome (eg, set of health care-associated infections), or defined as an adverse drug reaction or antibiotic associated diarrhoea without C. difficile laboratory/code confirmation.
Study population at risk from which cases were identified narrowed by a prior selection process (eg, only diarrheal patients, those on antibiotics, co-morbidity-specific groups).
Case-reports, systematic reviews, narrative reviews, letters to editors, brief communications, commentary pieces.


### Data extraction

Data from the included studies were extracted by at least two reviewers (EB, CL, JB, IL) using an Excel form which was piloted before its final use. Uncertainties or discrepancies on data extraction were resolved by discussion between extractors. We extracted data on the study setting, duration, and design, case ascertainment methods, and case definition details. Data were extracted in accordance to the following settings of CDI acquisition: health care facility-associated (HCF), hospital onset health care facility-associated (HO-HCF), community-associated (CA), any setting /unspecified CDI and for three selected high-risk settings: medical or general intensive care units (ICU), internal medicine wards (IM), and long-term care facilities (LTCF), including nursing homes. For ICD-coded hospitalizations, we included publications reporting the number of hospitalizations with CDI code as a primary and secondary diagnosis. or data extraction, incidence rates were collected based on number of CDI cases that adhered to recommended surveillance case definitions, but also included studies with modified case definitions if this was clearly reported. Authors of publications eligible for inclusion were contacted to obtain the number of cases if additional eligible categories were reported in order to enable inclusion in the meta-analysis. To minimize potential bias towards large studies in meta-analyses, data from multicentre publications were extracted per health care facility and counted as an individual data point. Studies with data from facilities also included in national surveillance or other multicentre publications were excluded to avoid potential duplication of the same study population. Depending on the case definition, incidence for the following metrics were extracted and standardized: number of CDI cases per admissions (per 1000 admissions per year), incidence density (per 10 000 patient-days), and cumulative incidence of CDI cases over the total population at risk (per 100 000 population per year).

### Statistical analysis

The total number of CDI cases by admissions, total patient-days, or population for each category, rates and 95% confidence intervals (CI) were extracted from publications as reported by authors or calculated based on available data. The distribution of CDI incidence rates identified was summarized in terms of interquartile ranges and median values as follows: for HCF and HO-HCF (including high-risk settings: ICU, IM, and LTCF) in terms of incidence density and cases per admissions, and for CA, any or unspecified setting of acquisition, or ICD-coded hospitalization in terms of cases per admissions and cumulative incidence.

Rates of CDI incidence were pooled using the *metan* command in Stata version 14 (StataCorp, College Station, Texas, USA). When a publication reported different rates for the same study population using different case ascertainment methods (eg, based on review of patients or laboratory charts vs administrative records) we used the number of CDI cases where patient’s records had been reviewed. A small value (0.05) was used as continuity correction to include rates with zero CDI cases in the meta-analysis. Estimates were developed using a random effects model, acknowledging the heterogeneity in observational studies conducted in diverse settings.

Where possible, we performed and reported subgroup meta-analyses by location (WHO regions; the Americas region was divided into North America and Latin America) and age group (children [≤15 years or pediatric hospitals], adults [≥15 years], elderly [≥65 years], and all or unspecified age [≥0-2 years and studies without information on age or where children were not explicitly excluded]). Subgroup analyses were performed for categories with at least three different data points.

## RESULTS

### Overview of included studies

**Figure 1:**
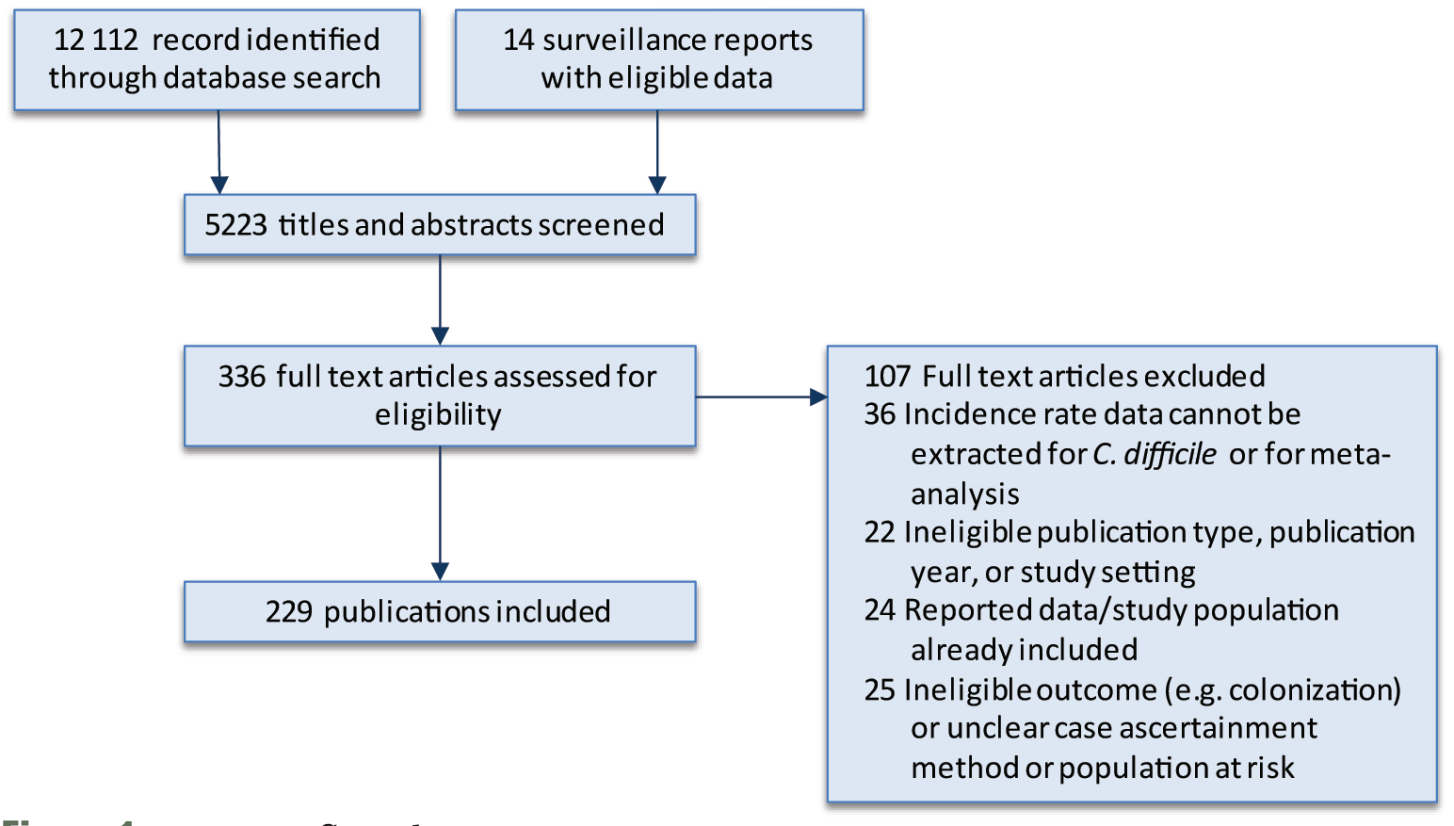
PRISMA flowchart.

A total of 229 publications [2,11,12,19-244], including 14 national surveillance reports, were included from over 12 000 publications assessed for eligibility. The literature review process is summarized in the PRISMA flowchart in **Figure 1**. Data extracted from each of the included studies are available in (Tables S2-S8 in **Online Supplementary Document**).

We included data on rates of CDI incidence from 41 countries. The majority of reports (195/229) were from Europe and North America. Incidence data eligible for inclusion from other regions: Western Pacific, Latin America, Eastern Mediterranean, and Africa were identified less frequently, and no reports from the South-East Asia region was included. Sub-analyses on CDI rates by world region are reported for Europe, North America and the Western Pacific regions for categories with suitable number of data points to conduct a meta-analysis.

Of the 229 publications included, 89 publications reported rates of HCF-CDI (incidence density and/or cases per admissions) and 57 publications reported data for HO-HCF CDI (incidence density and/or cases per admissions). Rates of CA-CDI were reported in 39 publications with almost all data from countries in North America, Europe, and the Western Pacific. CDI in high-risk settings (ICU, IM, and LTCF; 56 publications) were also for the most part identified from countries in North America, Europe, Latin America and the Western Pacific. CDI rates for children and the elderly were reported in 37 and 33 publications, respectively. The number of individual data on rates for each of the CDI categories and age groups are presented in **Table 2**.

**Table 2:** Number of data-points on CDI rates included in the review by category, age group, and world region

CDI	Hospital onset-health care facility associated				Intensive care unit				Internal Medicine			Healthcare facility-associated					Community-associated				Any setting						ICD-coded hospitalization			
	Children	Adults	Elderly	All ages	Any		Ward*		Any	Ward*		Children	Adults	Elderly	All ages	Children		Adults	Elderly	All ages	Children		Adult	Elderly	All ages		Children	Adults	Elderly	All ages
CDI per 1000 admissions:																														
Total (%)†	2 (5.5)	20 (54.1)	3 (8.2)	12 (32.5)		5 (31.3)	11 (68.8)	7 (63.7)		4 (36.4)	5 (10.7)		24 (51.1)	5 (10.7)	13 (27.7)	3 (11.6)		12 (46.2)	4 (15.4)	7 (27)		5 (4.7)	24 (22.5)	2 (1.9)	76 (71.1)	9 (27.3)		7 (21.3)	4 (12.2)	13 (39.4)
NA	1	14	2	7			5			1	2		15	3	3	1		10	3	2		3	16	2	5	9		7	4	10
EU		2		3		3	4	5		3	3		4	1	9	2		1	1	4		2	4		64					2
WP	1	3	1	1		1	2	1					4	1	1			1					3		3					1
LA				1		1		1					1							1					3					
AF		1																					1							
EM																									1					
	**CDI incidence density (per 10 000 patient-days)**															**CDI cumulative incidence (per 100 000 population per year)**														
Total *(%)*†	2 *(4.0)*	20 *(40.0)*	2 *(4.0)*	26 *(52.0)*		9 *(39.2)*	14 *(60.9)*	5 *(38.5)*		8 *(61.6)*	10 *(6.9)*		39 *(26.8)*	6 *(4.2)*	91 *(62.4)*	6 *(16.7)*		10 *(27.8)*	6 *(16.7)*	14 *(38.9)*		9 *(23.1)*	7 *(18.0)*	8 *(20.6)*	15 *(38.5)*	4 *(15.4)*		6 *(23.1)*	8 *(30.8)*	8 *(30.8)*
NA	1	15	2	16		1	7	1		2	6		21	2	12	5		6	3	6		4	3	3	4	4		6	7	6
EU				4		4	7	3		6	3		12	4	72	1		4	3	7		4	3	3	8				1	2
WP		3		6		3					1		4		5					1		1	1	1	3					
LA	1					1							1		1															
AF																														
EM		2						1					1		1															

CDI – *Clostridium difficile* infection, LTCF – long-term care facility, NA – North America, EU – Europe, WP – Western Pacific, LA – Latin America, AF – Africa, EM – Eastern Mediterranean, Any – health care facility and community-associated-CDI combined, also includes data from studies without distinction of case acquisition setting, ICD-coded – CDI hospitalization based on discharge code (ICD-CM 9 or ICM-10) for *C. difficile*, ICD – International Classification of Diseases

* Ward-onset.

†Percentage of data available by age group within category.

**Figure 2:**
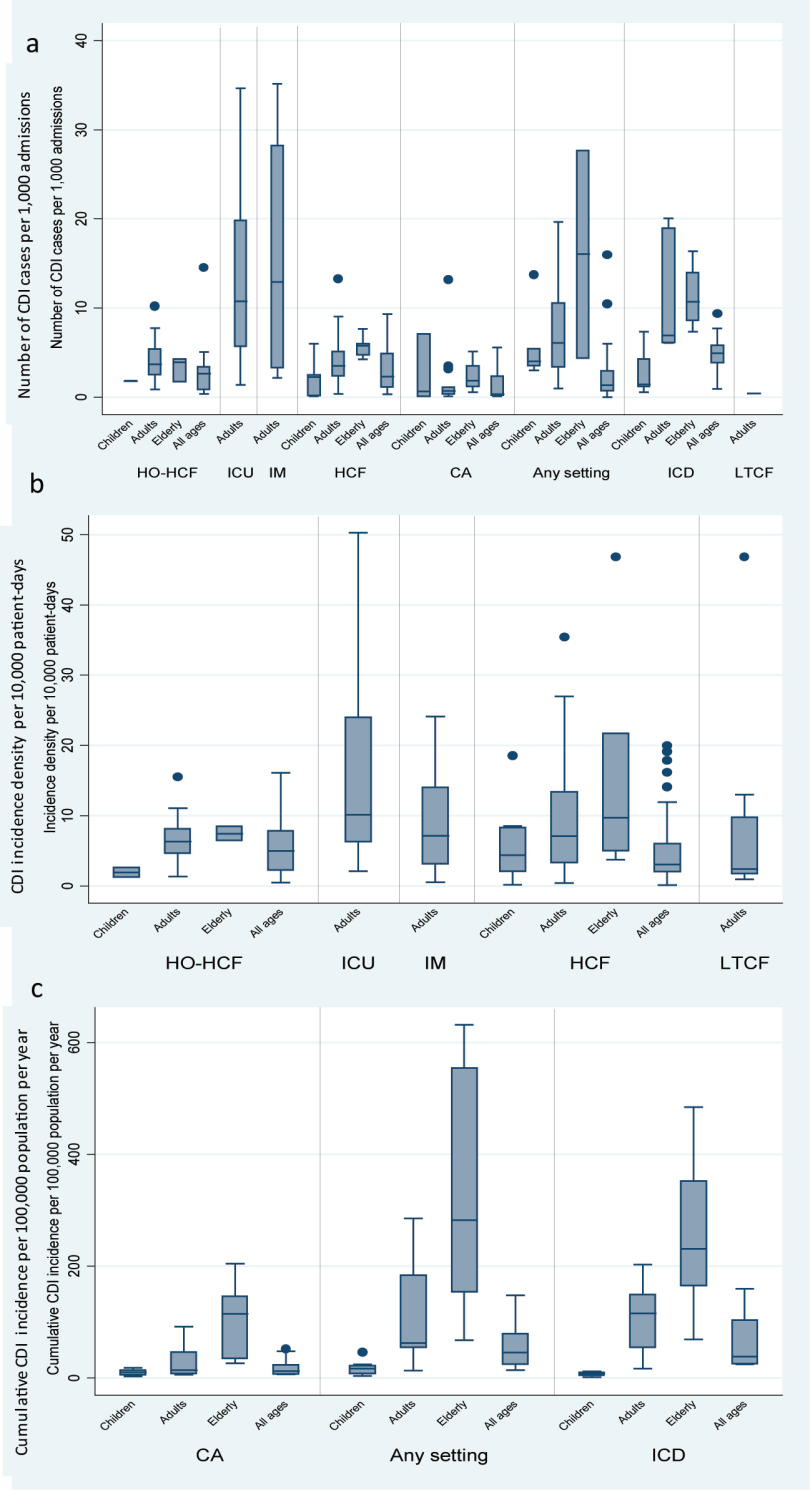
Distribution of CDI incidence rates by category and age groupI a) number of CDI cases per 1000 admissions per year, b) incidence density per 10 000 patient-days, c) cumulative incidence per 100 000 population per year. Abbreviations: CDI, *Clostridium difficile* infection; HO-HCF, hospital-onset health care facility-associated; ICU, intensive care unit; IM, internal medicine; HCF, health care facility-associated; CA, community-associated; ICD, international classification of diseases; LTCF, long term care facility.

Regardless of metric, there were large variations in rates of CDI incidence across categories and age groups. **Figure 2** depicts the median and range of all rates of CDI incidence reported in included studies in the review.

### Meta-analysis

**Table 3** shows the results from the meta-analyses, by CDI categories, regions and age groups.

**Table 3:** CDI incidence rates meta-estimates by category, age group, and region

CDI category	Age group	Overall		North America		Europe		Western Pacific		Other
**N**	**IR (95% CI)**	**N**	**IR (95% CI)**	**N**	**IR (95% CI)**	**N**	**IR (95% CI)**	**N**
**Number of CDI cases per 1000 admissions**										
**HO-HCF**	Children	2	1.78-1.80	1	1.78			1	1.80	
	Adults	20	3.58 (2.48-5.18)	14	4.27 (2.75-6.62)	2	1.50-4.22	3	3.05 (2.66-3.50)	1
	Elderly	3	3.05 (1.48-6.26)	2	1.69-4.29			1	3.93	
	All ages	12	2.09 (1.16-3.74)	7	2.60 (0.96-7.05)	3	0.98 (0.45-2.14)	1	2.68	1
**ICU**	Hospital	5	7.82 (3.81-16.05)	1	6.62	3	7.98 (1.72-36.98)			1
	Ward-onset	11	11.08 (7.19-17.08)	4	15.82 (8.75-28.59)	4	11.32 (5.24-24.46)	1		2
**IM**	Hospital	7	9.41 (3.95-22.40)			5	10.81 (3.88-30.14)	1	3.25	1
	Ward-onset	4	10.80 (3.15-37.06)	1	35.15	3	7.26 (1.57-33.74)			
**HCF**	Children	5	0.90 (0.17-4.80)	2	2.27-2.50	3	0.45 (0.3-8.01)			
	Adults	24	3.26 (2.37-4.50)	15	4.36 (3.01-6.31)	4	1.72 (0.89-3.32)	4	3.42 (2.74-4.27)	1
	Elderly	5	5.54 (4.81-6.38)	3	5.72 (4.29-7.63)	1	5.75	1	4.7	
	All ages	13	2.24 (1.66-3.03)	3	7.14 (4.26-11.98)	9	1.37 (1.06-1.79)	1	6.49	
**CA**	Children	3	0.52 (0.02-16.06)	1	0.66	2	0.03-7.09			
	Adults	12	0.60 (0.25-1.42)	10	0.77 (0.51-1.17)	1	0.26	1	0.12	
	Elderly	4	1.77 (0.52-6.04)	3	2.58 (1.21-5.51)	1				
	All ages	7	0.55 (0.13-2.37)	2	2.36-5.59	4	0.23 (0.18-0.29)			1
**Any**	Children	5	5.05 (3.12-8.17)	3	4.19 (3.28-5.34)	2	3.00-13.75			
	Adult	24	5.89 (4.64-7.50)	16	7.44 (5.62-9.85)	4	4.12 (0.84-20.40)	3	4.31 (3.02-6.15)	1
	Elderly	2	4.4-27.67	2	4.4-27.67					
	All ages	76	1.57 (1.34-1.85)	5	7.52 (4.00-14.13)	64	1.21 (0.98-1.49)	3	2.44 (0.80-7.42)	4
**ICD**	Children	9	2.01 (1.36-2.96)	9	2.00 (1.40-2.85)					
	Adults	7	9.64 (7.04-13.2)	7	9.69 (7.11-13.19)					
	Elderly	4	10.77 (7.98-14.54)	4	10.77 (7.98-14.54)					
	All ages	13	4.26 (3.61-5.05)	10	5.54 (4.69-6.54)	2	0.90-2.39	1	2.5	
**Incidence density per 10 000 patient-days**										
**HO-HCF**	Children	2	1.26-2.6	1	2.6			1	1.26	
	Adults	20	5.68 (4.91-6.56)	15	7.07 (6.09-8.20)			3	3.44 (2.49-4.76)	2
	Elderly	2	6.39-8.46	2	6.39-8.46					
	All ages	26	4.14 (3.10-5.53)	16	6.36 (5.53-7.19)	4	1.67 (0.58-4.84)	6	2.69 (1.32-5.49)	
**ICU**	Hosp	9	7.05 (4.42-11.24)	1	2.68	4	7.72 (3.72-16.00)	3	10.50 (7.57-14.56)	1
	Ward-onset	16	13.74 (9.46-19.93)	7	18.66 (13.04-26.69)	7	11.17 (5.44-22.93)	1		1
**IM**	Hosp	4	7.24 (3.57-14.71)	1	2.16	3	10.64 (5.02-21.78)			
	Ward-onset	8	9.21 (5.74-14.77)	2	16.00-24.13	6	7.13 (4.03-12.63)			
**HCF**	Children	10	3.53 (1.48-8.42)	6	7.43 (4.68-11.78)	3	0.88 (0.18-4.27)	1	2.00	
	Adults	39	5.78 (4.63-7.24)	21	10.25 (7.77-13.53)	12	2.30 (1.95-2.70)	4	5.30 (3.44-8.17)	2
	Elderly	6	10.95 (5.08-23.59)	2	11.76-46.83	4	7.48 (1.8-19.32)			
	All ages	91	3.54 (3.19-3.92)	12	7.03 (5.23-9.44)	72	3.14 (2.80-3.53)	5	3.45 (2.41-4.95)	2
**LTCF**	Ward-onset	8	4.41 (2.36-8.23)	7	5.52 (2.78-10.97)	1	0.94			0
**Cumulative incidence of CDI per 100 000 population**										
**CA**	Children	5	8.878 (5.67-13.88)	4	9.58 (5.96-15.41)	1	5.42			0
	Adults	9	19.95 (11.57-34.38)	6	25.45 (12.13-45.33)	3	14.36 (6.58-31.35)			0
	Elderly	6	74.41 (37.74-146.70)	3	83.67 (33.23-210.65)	3	65.69 (18.73-230.33)			0
	All ages	14	14.37 (9.92-20.82)	6	13.75 (7.9-23.95)	7	16.57 (8.80-31.22)	1	6.98	0
**Any**	Children	8	15.27 (7.72-30.192)	4	23.35 (15.08-36.14)	3	10.63 (3.04-37.17)	1	7.49	0
	Adults	7	78.95 (47.70-130.67)	3	89.57 (51.51-155.73)	3	77.25 (41.07-145.32)	1	58.93	0
	Elderly	7	323.32 (217.82-479.91)	3	502.04 (331.51-760.31)	3	272.44 (159.16-466.35)	1	142.76	0
	All ages	15	41.94 (30.30-58.05)	4	53.52 (24.08-118.98)	8	42.5 (26.94-66.92)	3	29.05 (21.61-37.33)	0
**ICD**	Children	4	5.45 (2.65-11.23)	4	5.45 (2.65-11.23)					0
	Adults	6	83.62 (64.72-108.04)	6	83.62 (64.71-108.04)					0
	Elderly	8	219.29 (187.33-256.69)	7	242.64 (207.01-284.39)	1	108			0
	All ages	8	49.36 (34.01-71.75)	6	59.91 (43.66-82.19)	2	32.70-23.60			0

CDI – *Clostridium difficile* infection, HO-HCF – hospital onset, health care facility-associated, ICU – intensive care unit, IM – internal medicine, HCF – health care facility-associated, CA – community-associated, ICD – International classification of diseases, LTCF – long term care facility, Other – Latin America, Africa, Eastern Mediterranean, N – Number of data entries in meta-analysis, IR – incidence rate point estimate; 95% CI – 95% confidence intervals

### CDI cases per admission

Incidence of CDI reported in individual studies ranged from 0 to 35.15 cases per 1000 admissions per year (**Figure 2**, panel a). The highest median number of CDI cases per admissions were reported from ICU and IM wards; with the highest meta-estimate for incidence rate (IR) of 11.08 (95% CI = 7.19-17.08) per 1000 admissions per year for ICU-onset CDI. Among all ages, meta-estimates rates of HO-HCF and HCF-CDI were similar (IR = 2.09 (95% CI = 1.16-3.74) per 1000 admissions per year and 2.24 (95% CI = 1.66-3.03) per 1000 admissions per year, respectively), and rates for CA-CDI were lower (IR = 0.55 (95% CI = 0.13-2.37) per 1000 admissions per year) (**Table 3**).

Estimates in the elderly were similar to those than for adults or higher compared to the other age groups (with children having the lowest incidence rates). Although regional level estimates were not strictly comparable, North America generally reported higher rates across CDI categories and age groups (**Table 3**).

### CDI incidence density

CDI incidence density reported in individual studies ranged from 0.11 to 50.3 per 10 000 patient-days (**Figure 2**, panel b). The highest estimated incidence density by meta-analysis was in the ICU and IM wards, the highest incidence rate 13.74 (95% CI = 9.46-19.93) per 10 000 patient-days was for ICU-onset CDI. The meta-analysis estimates for HO-HCF and HCF were similar (IR = 4.14 (95% CI = 3.10-5.53) per 10 000 patient-days and 3.54 (95% CI = 3.19-3.92) per 10 000 patient-days, respectively), as was the incidence density in LTCF (IR = 4.41 (95% CI = 2.36-8.23) per 10 000 patient-days) (**Table 3**).

Meta-estimates for the four age categories were calculated for HCF-CDI incidence density, where the highest density was observed among the elderly (IR = 10.95 (95% CI = 5.08-23.59) per 10 000 patient-days). Incidence density of HO-HCF CDI and HCF-CDI were also higher for adult populations compared to that in patients of all ages. Based on the limited data at regional level, we observed that North America had the highest CDI incidence density.

### Cumulative incidence of CDI

Cumulative incidence of CDI reported in individual studies ranged from 1.12 to 631.80 per 100 000 population per year (**Figure 2**, panel c). The majority of data for cumulative incidence of CDI hospitalizations were available from large databases in the United States. Based on data from ICD- coded hospitalizations, the estimate of cumulative incidence rate for all ages is 49.36 (95% CI = 34.01-71.75) per 100 000 population per year. This estimate is similar to the rate based on CDI cases identified through positive laboratory assay and clinical criteria (IR = 41.94 (95% CI = 30.30-58.05) per 100 000 population per year) (**Table 3**). For the latter, estimates from individual studies ranged from 13.42 per 100 000 population per year in a study conducted in Spain in 2003 where national laboratories were surveyed to 147.20 per 100 000 population per year in a study with sentinel surveillance in the United States where rates were adjusted for use of molecular laboratory assays. At the national level, increases in CDI hospitalization rates per 100 000 population were reported, for instance, in Finland (from 16.0 in 1996 to 34.0 in 2004) [12], in Belgium (from 16.5 in 1999 to 44.3 in 2008), and in the United States (from 48.8 in 1999 to 114.6 in 2008) [11].

Where meta-analysis by age groups was possible, the results for the cumulative incidence for the elderly was higher compared with the other age groups; for any CDI (regardless of setting of acquisition), the overall estimate from meta-analysis was 323.32 (95% CI = 217.82-479.91) per 100 000 population per year. Europe had the highest estimates of cumulative incidence rate CA-CDI in all age groups, though confidence intervals overlap with the estimate for North America.

## DISCUSSION

The aim of this study was to examine reports of CDI incidence rates in order to develop an overall estimate and to identify gaps in the current evidence base globally. We found variations of CDI occurrence in terms of rates, within categories as well as between world regions. From our meta-analyses, the estimated overall incidence rate of HCF-CDI for patients of all ages was 2.24 per 1000 admissions per year and 3.54 per 10 000 patient-days.

The implementation of robust surveillance systems is important to ascertain the burden of CDI across and to help identify any future changes in rates [2,14]. Through our review of the literature, we found that the highest rates of CDI for the majority of categories assessed were reported in North America. It is likely that this finding is due to improvements in case detection and influenced by the use of high-sensitivity testing methods such as nucleic acid amplification testing which can lead to overestimation of CDI rates [2,149,245]. In Europe, our meta-estimate for health care-associated CDI density rates (3.14 [95%CI = 2.80-3.53] per 10 000 patient-days) is similar to estimates from regional surveys in 2005, 2008 and 2012-13 [3-5] (for which the overall estimate is 4.08 (95%CI = 3.52-4.74) per 10 000 patient days when data are analyzed by meta-analysis, (Appendix S9 in **Online Supplementary Document**). Our meta-estimate is also similar to the median rate based on aggregate data from 37 European hospitals in 14 countries in 2013 (median 3.7 (range: 0.6-18.5) per 10 000 patients days) [246]. Variations in CDI rates may also be due to other differences in case ascertainment, both regionally and globally, such as practices and criteria for specimen collection, testing policies and methodologies, under-ascertainment of cases, and reporting requirements [5,246,247]. Efforts to harmonize CDI surveillance protocols within and across countries, such as in Europe, will facilitate the monitoring of epidemiological changes, implementation of infection control protocols [246,248]. Our review shows there is a paucity of data on the incidence of CDI from regions other than North America, Europe and the Western Pacific. This gap in the evidence base is noteworthy as there is evidence of high burden of CDI in other world regions [249]. Limited country-specific data may be due to a combination of lower prevalence and *C. difficile* not being tested commonly in these regions [250,251]. Identifying the global transmission of *C. difficile* by molecular characterization is also an important component of surveillance [252] that will further advance our understanding of its associated burden.

Our assessment of rates by age groups shows the importance of age-specific rates to monitor and address the burden of CDI effectively. Our meta-estimates of CDI rates provide evidence for the magnitude of the burden among across age groups and consistently show that elderly populations are disproportionally affected by CDI; as incidence rates increase with increasing age [11] and over 80% of CDI deaths occur in those over 65 years [2]. The high burden of *C. difficile* on older adults may be due to a range of factors including: more frequent interactions with health care systems, higher use of antimicrobials, and physiologic changes such as decreases in immune responses and multi-morbidity [2]. Some risk factors for CDI in the elderly are modifiable, through actions such as antimicrobial stewardship [253] and the targeting of severe and recurrent cases of CDI, which may impact morbidity and outcomes [254]. These approaches are also relevant to cases of CDI in other age groups. Though we found low rates of CDI among the paediatric population, we also identified discrepancies in the inclusion criteria for classifying pediatric CDI cases. There is a high carriage rate of *C. difficile* in neonates. Consequently, *C. difficile* is often thought to be non-pathogenic in infants and assessing the burden of CDI in this population is more difficult [255]. Further, episodes of disease are often of shorter duration and fewer complications as compared to adults [256]. Considering that increases in the rate of CDI hospitalizations in children have been reported, in North America for instance [244,257,258], and the limited information regarding the burden of CDI, careful assessment of surveillance data among the pediatric population is warranted. It is important that studies adhere to standardized surveillance recommendations (eg, infants should be excluded both from the number of cases and the study population in incidence rate calculations) and that CDI rates are analyzed for key age groups to identify monitor subgroups at risk of disease.

While there is evidence from individual studies that the incidence of CDI in ICU patients is decreasing [37,112], our study showed CDI rates were consistently high in the ICU or IM setting. These high rates may be related to the multiple major risk factors that are often found in critically ill or older hospitalized patients [259]. CDI in the ICU has been associated with an increase in mortality, and increased length of stay compared to other settings [259,260] highlighting the importance for developing strategies and protocols for reducing infection in these settings. Our review also indicates the need for assessments of CDI in nursing homes and other LTCF, where limited data are available currently and the burden is estimated to be high [222,261,262].

The rates of CA-CDI identified through this analysis were generally low, yet, comparable across world regions. Under-ascertainment or under-reporting of CA-CDI may have contributed to the lower rates identified, as well as the lower density of individuals in the community who are at high-risk for CDI. In countries where research has been conducted, such as in the US and the UK, a substantial proportion of CDI cases that are acquired and have symptom onset in the community (20%-45% of all CDI are CA-CDI [1,2,75,263]). Understanding the burden of CA-CDI is important as these cases may be characterized by different risk factors, with a younger age group, presenting with less severe disease, and with less prior exposure to antimicrobials [121]. Further knowledge of these factors and on the links with health care setting is important to develop strategies for lowering the rate of CDI in these distinct populations.

To our knowledge, this is the largest meta-analysis of CDI incidence data to date obtained data using publications from a 10-year period to identify a substantial amount of data. This comprehensive review allowed us to estimate CDI rates for a range of metrics across settings. Through the exclusion of studies that reported rates from outbreaks, our estimates provide a baseline epidemiological rate of the burden associated with CDI. However, there are limitations to our review and meta-analysis. One limitation is the low number of reports from several countries, preventing a truly global picture of CDI rates. Though this could partially be influenced by our inclusion criteria (peer-reviewed articles in English and Spanish), we added to the global reach of our search by including data from publically available surveillance reports. Other sources of data, beyond the scope of our study, including short bulletins and publications in other languages need to be systematically assessed. We also identified a limited number of reports of CDI for some clinical settings and case definitions (such as LTCF and community), which limits the estimation of the impact of *C. difficile* on these diverse populations. We used data in studies published between 2005 and 2015 to include a wide range of relevant reports. However, rates in the early years of these publications (ranging from 1993 until 2015) may be less representative of the current epidemiology of CDI due to changes in rates over time. In addition, some recent data on CDI incidence from 2015 may have not been included in our estimates because our search criteria might not have captured studies during this year. Finally, results from meta-analyses are impacted by the characteristics of the primary studies, which may include variations in case definitions, testing methods, and case identification. We aimed to limit these variations in this study through our inclusion criteria and analysis, yet, heterogeneity remains.

## CONCLUSIONS

This study provides baseline epidemiological CDI rates and uncertainty ranges for a variety of settings highlighting the high global burden of disease associated with *C. difficile*. Our results also emphasize the need for the application of standardized surveillance recommendations, the reporting of age-specific incidence rates, and research in countries were sustainable surveillance by national or regional authorities is not possible to obtain a fuller picture of the burden of CDI. Improving surveillance and case ascertainment rates globally is important to characterize, to understand the burden of disease associated with *C. difficile*, and to implement effective prevention and infection control measures.

## Additional material

Online Supplementary Document
