# Supplementary material for: Global burden of Clostridium difficile infections: a systematic review and meta-analysis
Source: J Glob Health. 2018 Dec 21;9(1):010407. doi: 10.7189/jogh.09.010407 (PMC6304170; doi:10.7189/jogh.09.010407)

Supplementary Material: Balsells et al. Global burden of Clostridium difficile infections: a systematic review and meta-analysis

Supplementary Table 1. Search strategies by database

	**Medline In-Process & Other Non-Indexed Citations and Ovid MEDLINE(R) 1946 to Present**
1	exp Clostridium difficile/
2	exp Enterocolitis, Pseudomembranous/
3	("difficile" and (infection or colitis or diarrh?ea)).mp.
4	1 or 2 or 3
5	incidence.mp. or exp Incidence/ or incidence studies/
6	epidemiology.mp. or exp Epidemiology/
7	burden.mp.
8	5 or 6 or 7
9	4 and 8
10	limit 9 to (humans and yr="2005 -Current")
	**Embase (Ovid)**
1	exp Peptoclostridium difficile/
2	exp Clostridium difficile infection/
3	exp pseudomembranous colitis/
4	("difficile" and (infection or colitis or diarrh?ea)).mp.
5	1 or 2 or 3 or 4
6	exp incidence/ or incidence.mp.
7	epidemiology/ or epidemiology.mp.
8	burden.mp.
9	6 or 7 or 8
10	5 and 9
11	Limit 10 to (human and yr="2005 -Current")
	**Global Health (Ovid)**
1	clostridium difficile.mp. or exp Clostridium difficile/
2	pseudomembranous colitis.mp.
3	"clostridium difficile infection".mp.
4	1 or 2 or 3
5	incidence.mp. or exp incidence/
6	exp epidemiology/ or epidemiology.mp.
7	burden.mp.
8	5 or 6 or 7
9	4 and 8
10	Limit 9 to yr=”2005-Current”
	**CINAHL Plus**
S1	(MH "Clostridium Difficile") OR "clostridium difficile" OR (MH "Clostridium Infections") OR (MH "Enterocolitis, Pseudomembranous")
S2	(MH "Incidence") OR "incidence"
S3	(MH "Epidemiology+") OR "epidemiology"
S4	"burden"
S5	S2 OR S3 OR S4
S6	S1 AND S5
S7	Limiters: Publication Year: 2005-2016
	**Global Health Library WPRIM, IMSEAR, EMEMR and LILACS**
	(clostridium difficile) AND (burden or epidemiology or incidence)
	**Web of Science:**
	TI=(clostridium difficile) AND TS=(incidence or prevalence or epidemiology or burden)

Grey literature

To identify unpublished sources, a targeted-internet search was conducted for a review on CDI infection control and prevention guidelines (Balsells E, et al. J Glob Health. 2016 Dec;6(2):020410). Briefly, two reviewers conducted an internet search (with the Google search engine) of publically available national or organisational guidelines, related to CDI control (published between 2000 and 2015 and for any healthcare setting). We also accessed individual national departments/ministries of health and the websites globally of professional societies including those members of the International Federation of Infection Control. When national surveillance reports were identified in websites, these were included in this study if they met inclusion criteria.

Supplementary Table 2. HCF-CDI incidence density and number of cases per admissions (all ages and adults)

Region	Reference	Country	Study yrs (if <1 year: mo/wk)	Age (years)	CDI ascertainment	Laboratory confirmation	HCF-CDI Incidence Rate (95% Confidence Interval)			
							Parameters	Cases	10,000 patient-days	1,000 admissions
EM	Al-Tawfiq (2010)	S. Arabia	2007-08	All ages	Lab+symp	ELISA Tox A&B	All CDI IP cases	42	2.04 (1.51-2.76)	
EU	Starzengruber (2014)	Austria	2012	No details	Lab+symp	GDH EIA, PCR	All CDI IP cases	278	5.23 (4.65-5.88)	
EU	Ahmetagic (2013)	Bosnia	2009-12	≥ 2	Lab+symp	Tox A/B (ELISA)	>48 hours/IP<4 wks	347	2.23 (1.97-2.52)	1.57 (1.39-1.77)
EU	Ivanova (2011)	Bulgaria	2008-10	> 2	Lab+symp (i)	EIA ToxA&B, Cul	All CDI IP cases	15	0.34 (0.20-0.56)	
EU	Novak (2014)	Croatia	2010-11	All ages	Lab+symp (i)	EIA A&/B	IP<4 wks	36	0.42 (0.30-0.58)	0.34 (0.25-0.47)
EU	Kanerva (2013)	Finland	2008-10	≥0	Lab+symp (IP)	Tox A/B, Cul, PCR	Nosocomial	1976	2.83 (2.71-2.96)	
EU	Barbut (2007)	France	2000-04	All ages	Lab+symp	Cyt, Cul, EIA, PCR	All CDI IP cases	151	1.40 (1.19-1.64)	0.87 (0.72-1.04)
EU	Eckert (2013)	France	2009 (6m)	No details	V-Survey Lab+symp	EIA Tox, Cyt, PCR, Cul, GDH	>48 hours/IP<4 wks	763	1.33 (1.24-1.43)	0.64 (0.59-0.68)
EU	Gastmeier (2009)	Germany	2007	No details	Surveillance	Tox, Cul	>48 hours/IP<4 wks	2213	4.82 (4.62-5.04)	3.39 (3.25-3.53)
EU	KISS (2015)	Germany	2008-14	All ages	Surveillance	No details	>3 days/IP<4wks	57974	4.23 (4.19-4.26)	
EU	Mattner (2008)	Germany	2006	No details	Lab	ELISA	All IP cases	400	10.8 (133.76-11.91)	
EU	Meyer (2012)	Germany	2010	All ages	Surveillance	Tox, Cul	>48 hours/IP<4 wks	5183	4.65 (4.53-4.78)	3.37 (3.28-3.47)
EU	HPSC (2013)	Ireland	2013	≥2	Lab+symp (i)	GDH, Tox A&B, PCR	>48 hours/IP<4 wks	4759	2.48 (2.41-2.55)	
EU	Hensgens (2013)	Netherlands	2006-09	All ages	Lab+symp (i)	ELISA Tox A&/B, Cyt	>48 hours/IP<12w	1175	1.81 (1.71-1.91)	1.14 (1.00-1.12)
EU	Kooi [01] (2008)	Netherlands	2004-05	No details	Lab only	Tox	All CDI IP cases	34	3.28 (2.34-4.59)	
EU	Kooi [02] (2008)	Netherlands	2004-05	No details	Lab only	Tox	All CDI IP cases	58	2.98 (2.3-3.86)	
EU	Kooi [03] (2008)	Netherlands	2004-05	No details	Lab only	Tox	All CDI IP cases	136	4.85 (4.1-5.74)	
EU	Kooi [04] (2008)	Netherlands	2004-05	No details	Lab only	Tox	All CDI IP cases	50	3.96 (3-5.22)	
EU	Kooi [05] (2008)	Netherlands	2004-05	No details	Lab only	Tox	All CDI IP cases	83	6.01 (4.85-7.45)	
EU	Kooi [06] (2008)	Netherlands	2004-05	No details	Lab only	Tox	All CDI IP cases	29	2.66 (1.85-3.83)	
EU	Kooi [07] (2008)	Netherlands	2004-05	No details	Lab only	Tox	All CDI IP cases	24	1.25 (0.84-1.87)	
EU	Kooi [08] (2008)	Netherlands	2004-05	No details	Lab only	Tox	All CDI IP cases	21	0.66 (0.43-1.01)	
EU	Kooi [09] (2008)	Netherlands	2004-05	No details	Lab only	Tox	All CDI IP cases	65	1.6 (1.26-2.04)	
EU	Kooi [10] (2008)	Netherlands	2004-05	No details	Lab only	Tox	All CDI IP cases	94	4.72 (3.86-5.78)	
EU	Kooi [11] (2008)	Netherlands	2004-05	No details	Lab only	Tox	All CDI IP cases	34	1.65 (1.18-2.31)	
EU	Kooi [12] (2008)	Netherlands	2004-05	No details	Lab only	Tox	All CDI IP cases	30	1.96 (1.37-2.8)	
EU	Kooi [13] (2008)	Netherlands	2004-05	No details	Lab only	Tox	All CDI IP cases	109	5.87 (4.86-7.09)	
EU	Kooi [14] (2008)	Netherlands	2004-05	No details	Lab only	Tox	All CDI IP cases	15	1.7 (1.03-2.82)	
EU	Kooi [15] (2008)	Netherlands	2004-05	No details	Lab only	Tox	All CDI IP cases	20	1.74 (1.12-2.7)	
EU	Kooi [16] (2008)	Netherlands	2004-05	No details	Lab only	Tox	All CDI IP cases	1	0.11 (0.02-0.78)	
EU	Kooi [17] (2008)	Netherlands	2004-05	No details	Lab only	Tox	All CDI IP cases	33	1.92 (1.37-2.7)	
EU	Kooi [18] (2008)	Netherlands	2004-05	No details	Lab only	Tox	All CDI IP cases	65	3.66 (2.87-4.67)	
EU	Kooi [19] (2008)	Netherlands	2004-05	No details	Lab only	Tox	All CDI IP cases	9	1 (0.52-1.92)	
EU	Kooi [20] (2008)	Netherlands	2004-05	No details	Lab only	Tox	All CDI IP cases	60	2.07 (1.61-2.67)	
EU	Kooi [21] (2008)	Netherlands	2004-05	No details	Lab only	Tox	All CDI IP cases	44	8.26 (6.15-11.1)	
EU	Kooi [22] (2008)	Netherlands	2004-05	No details	Lab only	Tox	All CDI IP cases	16	0.52 (0.32-0.85)	
EU	Kooi [23] (2008)	Netherlands	2004-05	No details	Lab only	Tox	All CDI IP cases	30	1.39 (0.97-1.99)	
EU	Kooi [24] (2008)	Netherlands	2004-05	No details	Lab only	Tox	All CDI IP cases	44	1.77 (1.32-2.38)	
EU	Paltansing [01] (2007)	Netherlands	2005	All ages	Lab+symp (i)	Tox A&B EIA	All CDI IP cases	11	6 (3.32-10.84)	
EU	Paltansing [02] (2007)	Netherlands	2005	All ages	Lab+symp (i)	Tox A&B EIA	All CDI IP cases	13	4 (2.32-6.89)	
EU	Paltansing [03] (2007)	Netherlands	2005	All ages	Lab+symp (i)	Tox A&B Cyt	All CDI IP cases	3	2 (0.65-6.2)	
EU	Paltansing [04] (2007)	Netherlands	2005	All ages	Lab+symp (i)	Tox A&B Cyt	All CDI IP cases	6	3 (1.35-6.68)	
EU	Paltansing [05] (2007)	Netherlands	2005	All ages	Lab+symp (i)	Tox A&B EIA	All CDI IP cases	4	2 (0.75-5.33)	
EU	Paltansing [06] (2007)	Netherlands	2005	All ages	Lab+symp (i)	Tox A&B Cyt	All CDI IP cases	7	3 (1.43-6.29)	
EU	Paltansing [07] (2007)	Netherlands	2005	All ages	Lab+symp (i)	Tox A&B Cyt	All CDI IP cases	3	2 (0.65-6.2)	

Region	Reference	Country	Study yrs (if <1 year: mo/wk)	Age (years)	CDI ascertainment	Laboratory confirmation	HCF-CDI Incidence Rate (95% Confidence Interval)			
							Parameters	Cases	10,000 patient-days	1,000 admissions
EU	Paltansing [8] (2007)	Netherlands	2005	All ages	Lab+symp (i)	Tox A&B EIA	All cases IP	1	<1	
EU	Paltansing [9] (2007)	Netherlands	2005	All ages	Lab+symp (i)	Tox A&B EIA + Cyt	All cases IP	4	<1	
EU	Paltansing [10] (2007)	Netherlands	2005	All ages	Lab+symp (i)	Tox A&B Cyt	All cases IP	6	2 (0.9-4.45)	
EU	Paltansing [11] (2007)	Netherlands	2005	All ages	Lab+symp (i)	Tox A&B Cyt	All cases IP	4	2 (0.75-5.33)	
EU	Paltansing [12] (2007)	Netherlands	2005	All ages	Lab+symp (i)	Tox A&B Cyt	All cases IP	1	1 (0.14-7.1)	
EU	Paltansing [13] (2007)	Netherlands	2005	All ages	Lab+symp (i)	Tox A&B EIA	All cases IP	4	2 (0.75-5.33)	
EU	RIVM	Netherlands	2012-14	≥2	S-Surv (IP)	EIA, EIA+GDH, LAMP, PCR	All cases IP	2412	2.93 (2.81-3.05)	1.55 (1.47-1.63) N: 1480(2012-13
EU	Pituch (2015) [1]	Poland	2011-13	≥2	Lab+symp	EIA TOX A/B, TCa	All cases IP	55	3 (4.44-3.91)	
EU	Pituch (2015) [2]	Poland	2011-13	≥2	Lab+symp	GDH+TOX A/B	All cases IP	263	14.1 (100.34-15.91)	
EU	Pituch (2015) [3]	Poland	2011-13	≥2	Lab+symp	EIA TOX A/B	All cases IP	190	9.63 (0.33-11.11)	
EU	Pituch (2015) [4]	Poland	2011-13	≥2	Lab+symp	EIA GDH and EIA TOX A/B; TC, PCRa	All cases IP	193	5 (0.27-5.76)	
EU	Pituch (2015) [5]	Poland	2011-13	≥2	Lab+symp	GDH+TOX A/B	All cases IP	133	3.37 (0.3-3.99)	
EU	Pituch (2015) [6]	Poland	2011-13	≥2	Lab+symp	GDH+TOX A/B	All cases IP	95	2.37 (0.25-2.89)	
EU	Pituch (2015) [7]	Poland	2011-13	≥2	Lab+symp	GDH+TOX A/B, TCa	All cases IP	190	2.83 (0.06-3.27)	
EU	Pituch (2015) [8]	Poland	2011-13	≥2	Lab+symp	EIA TOX A/B, TCa	All cases IP	868	19.13 (0.39-20.45)	
EU	Pituch (2015) [9]	Poland	2011-13	≥2	Lab+symp	EIA GDH and EIA TOX A/B, TCa	All cases IP	192	3 (7.71-3.46)	
EU	Pituch (2015) [10]	Poland	2011-13	≥2	Lab+symp	GDH+TOX A/B	All cases IP	519	7.2 (2.69-7.85)	
EU	Pituch (2015) [11]	Poland	2011-13	≥2	Lab+symp	GDH+TOX A/B, TCa	All cases IP	297	19.97 (1.31-22.36)	
EU	Pituch (2015) [12]	Poland	2011-13	≥2	Lab+symp	EIA TOXA/B or qPCR; TCa	All cases IP	1407	16.13 (4.41-17.01)	
EU	Pituch (2015) [13]	Poland	2011-13	≥2	Lab+symp	GDH+TOX A/B, Illuminigenea	All cases IP	703	6.47 (13.71-6.97)	
EU	Reigadas (2015)	Spain	2013	≥2	Lab+symp	Tox A/B Cul PCR	>48 hours/IP<4 wks	152	8 (2.15-9.38)	
EU	Rodriguez-Pardo (2013)	Spain	2009	All ages	Lab+symp	Tox A&B, Cul	All cases IP	365	1.93 (1.74-2.14)	
EU	Siller-Ruiz (2014)	Spain	2011-12	≥2	Lab+symp (i)	Tox A&B, Cul, Cyt	>48 hours/IP<4 wks	41	1.15 (0.85-1.56)	
EU	Folkhälsomyndigheten	Sweden	2014	≥2	Surv, Lab only	No details	All CDI IP cases	7691	11 (10.76-11.25)	
EU	Fenner (2008)	Switzerland	2006-07	All ages	Lab only (i)	EIA ToxA/B	All CDI IP cases	78	2.3 (1.84-2.87)	
EU	Kohler (2013)	Switzerland	2009-10	No details	Same facility	Cyt	>48 hours/IP<4 wks	108	2.3 (1.90-2.77)	
EU	PHE (M-Surv)	UK England	2006-14	≥2	Lab+symp	No details	All cases IP	199146	6.98 (6.95-7.01)	
EU	Aldeyab (2009)	UK Northern Ireland	2002-07	≥2	Lab+symp	Tox A&B	All cases IP	393	6.00 (5.44-6.62)	
EU	HSC (2011-15)	UK Northern Ireland	2011-15	≥2	Lab+symp	Tox A&B	All cases IP	2477	2.65 (2.55-2.76)	
EU	Jen (2012)	UK-England	1997-10	≥2	ICD10		IP+≥72LOS	205037	3.53 (6.64-3.54)	2.3 (2.29-2.31)
EU	Reddy (2010)	UK-Scotland	2003-07	All ages	Lab		All IP new cases	4922	1.69 (2.32-1.74)	
LA	Ministry of Health	Uruguay	2012	≥2	S-Surv, Lab only	No details	IP<4 wksks	153	3.0 (2.56-3.52)	
NA	Lambert (2009)	Canada	2005-06	All ages	Lab+symp	EIA GDH, Tox A, Cyt	>48 hours/IP<4 wks	646	9.11 (8.44-9.84)	
NA	Longtin (2013)	Canada	2010-11	All ages	Lab+symp	PCR, EIA/Cyt	>72 hours/IP<4 wks	85	8.88 (1.55-10.98)	
NA	PHA (2014)	Canada	2007-12	≥1	Lab+symp	ToxA/B, Cul	>72 hours/IP<4 wks	18871	6.46 (6.36-6.55)	4.88 (4.81-4.95)
NA	Benoit (2011)	US	2007-08	All ages	Lab only	EIA	>72 hours/IP 30 days	2904	10.5 (10.2-10.9)	
NA	Catanzaro (2012)	USA	2009-10	No details	Lab only	EIA, Cyt, GDH, Cul	All CDI IP cases	57	11.94 (9.21-15.47)	
NA	Cloud (2009)	US	2004-06	No details	Lab+symp	Cul, PCR	Nosocomial	272	8.66 (7.69-9.75)	
NA	Kanamori (2015)	US	2003-12	No details	Lab	No details	All CDI IP cases	887	3.97 (3.72-4.24)	

Region	Reference	Country	Study yrs (if <1 year: mo/wk)	Age (years)	CDI ascertainment	Laboratory confirmation	HCF-CDI Incidence Rate (95% Confidence Interval)			
							Parameters	Cases	10,000 patient-days	1,000 admissions
NA	Kang (2014)	US	2005-11					685	4.33 (7.16-4.67)	
NA	Khanna (2012)	US	1991-05	All ages	ICD9+Chart review	ICD9 (I/OP)+Chart (Cyt,EIA)	>48 hours/IP<4 wks	192	1.66 (1.44-1.91)	
NA	King (2011)	US	2009	No details	Lab+symp	ToxA&B	>48 hours/IP<4 wks	89		8.08 (6.56-9.95)
NA	Montoya (2013)	US	2011	All ages	Lab+symp	Tox, PCR	>48 hours/IP<4 wks	54	4.5 (3.45-5.88)	
NA	Sohn [X] (2005)	US	2000-03	No details	Lab+symp	Unknown	>48 hours/IP<4 wks	294	11.33 (10.11-12.70)	
NA	Tartof (2014)	US	2011-12	All ages	Lab only	PCR	>48 hours/IP<4 wks	2505	17.84 (17.16-18.55)	9.32 (8.97-9.70)
WP	Mitchell (2011)	Australia	2006-10	≥ 2	Lab+symp	EIA, Cul	All CDI IP cases	357	3.35 (3.02-3.72)	
WP	Slimings (2014)	Australia	2011-12	≥ 2	N-Surv, Lab+symp	Tox A/B, PCR	>48 hours/IP<4 wks	4446	2.95 (2.86-3.04)	
WP	Van Gessel (2008)	Australia (W)	2006	≥ 2	Lab+symp (i)	EIA, Cyt ToxA/B, Cul	>72 hours/ IP<4 wks	46	1.2 (0.9-1.6)	
WP	Kim (2012)	S. Korea	2009-10	No details	Lab+symp	PCR, Tox A&B	>72 hours/ IP<8 wks	166	7.16 (6.15-8.34)	6.49 (5.57-7.56)
WP	Koh (2007)	Singapore	2002-03 (5m)	All ages	Lab+symp (IP)	EIA A&B, Cul	All CDI IP cases	88	5.38 (4.37-6.63)	
	**Adults**									
EM	Khan (2012)	Qatar	2006-09	≥15	Lab+symp	EIA, A/B	72h/IP<2 months	123	1.6 (1.34-1.91)	
EU	Wenisch (2012)	Austria	2009-10	Adult	Lab+symp	ELISA ToxA&B+PCR	>48 hours/IP<4 wks	133	5.65 (4.77-6.70)	
EU	Balihar (2014)	Czech Rep	2006-08	≥18	Lab+symp	ELISA, EIA ToxA/B	>72 hours/IP 7 days	156	1.01 (0.86-1.18)	0.83 (0.71-0.97)
EU	Vesteinsdottir (2012)	Iceland	2010-11	≥18	Lab only	ELISA ToxA/B	>72 hours/IP 6 wks	59		1.97 (1.53-2.54)
EU	Di Bella [1] (2013)	Italy	2006-11	≥18	Lab only	EIA Tox A&B	All cases IP	70	1.44 (1.14-1.82)	
EU	Di Bella [2] (2013)	Italy	2006-11	≥18	Lab only	EIA Tox A&B	All cases IP	69	1.01 (0.80-1.28)	
EU	Di Bella [3] (2013)	Italy	2006-11	≥18	Lab only	EIA Tox A&B	All cases IP	77	0.39 (0.31-0.49)	
EU	Di Bella [4] (2013)	Italy	2006-11	≥18	Lab only	EIA Tox A&B	All cases IP	138	1.67 (1.41-1.97)	
EU	Di Bella [5] (2013)	Italy	2006-11	≥18	Lab only	EIA Tox A&B	All cases IP	48	1.62 (1.22-2.15)	
EU	Laza (2015)	Romania	2013-14	≥18	Lab+symp	EIA Tox A&B	All cases IP	219	17.16 (15.03-19.6)	
EU	Mertz (2010)	Switzerland	2004-08	Adults	Lab only	EIA ToxA&/B, Cul	All cases IP	405	2.30 (2.09-2.54)	
EU	Ergen (2009)	Turkey	2004-05	≥18	Lab+symp	Tox A&B, Cul	Nosocomial	19	2.60 (1.66-4.08)	2.10 (1.34-3.29)
EU	Jen (2012)	UK-England	1997-10	≥20	ICD10+≥72h LOS	No details	All cases IP	203488	3.64 (3.63-3.66)	2.58 (2.57-2.59)
EU	HPS (09-14)	UK-Scotland	2009-14	≥15	Lab		All cases IP	14045	4.42 (4.34-4.49)	
LA	Silva (2013)	Brazil	2005-09	>18	Lab+symp	EIA Tox A&B	>72 hours/IP<72 h	205	3.78 (3.30-4.34)	1.44 (1.26-1.65)
NA	Gravel (2009)	Canada	2004-05 (6m)	≥18	Lab+symp	ToxA/B, Cul	>72 hours/IP<2 mo	1430	6.46 (6.14-6.81)	4.64 (4.41-4.89)
NA	PHA (2014)	Canada	2007-12	≥18	Lab+symp	ToxA/B, Cul, PCR	>72 hours/IP< 1 mo	18291	6.56 (6.47-6.66)	5.06 (4.99-5.14)
NA	Armbruster (2012)	US	2005-09	Army staff	Lab only	No details	IP<12 wks	165		2.34 (2.01-2.73)
NA	Bengualid (2011)	US	2006-08	≥18	Lab+symp	EIA Tox A&B	>72 hours/IP<6 months	302	7.93 (7.08-8.88)	
NA	Dubberke [A] (2010)	US	2000-06	≥18	Lab only	EIA Tox	>48 hours/IP<4 wks	2888	18.12 (17.47-18.79)	9.06 (8.73-9.39)
NA	Dubberke [B] (2010)	US	2000-06	≥18	Lab only	EIA Tox	>48 hours/IP<4 wks	414	11.94 (10.85-13.15)	5.83 (5.30-6.42)
NA	Dubberke [C] (2010)	US	2000-06	≥18	Lab only	EIA Tox	>48 hours/IP<4 wks	629	4.52 (4.18-4.89)	2.48 (2.29-2.68)
NA	Dubberke [D] (2010)	US	2000-06	≥18	Lab only	EIA Tox	>48 hours/IP<4 wks	478	7.07 (6.46-7.73)	4.21 (3.85-4.60)
NA	Dubberke [E] (2010)	US	2000-06	≥18	Lab only	EIA Tox	>48 hours/IP<4 wks	700	5.52 (5.12-5.94)	4.31 (4.00-4.64)
NA	Gase (2013)	US	2009 (6m)	≥18	Lab+symp (Aud)	EIA Tox A&B	>72 hours/IP<4 wks	1891	10.18 (9.73-10.65)	5.42 (5.18-5.67)
NA	Gase (2013)	US	2009 (6m)	≥18	Lab only (LabID)	EIA Tox A&B	>72 hours/IP<4 wks	2234	12.03 (11.55-12.54)	6.4 (6.14-6.67)
NA	Gutierrez (2013)	US	1998-10	Army staff	ICD9+Lab	No details	IP<12 wks	1405		1.30 (1.23-1.37)
NA	Kutty (2008)	US	2005	≥18	Lab+symp	EIA Tox A&B	>48 hours/IP<4 wks	516	10.16 (9.32-11.07)	
NA	Gordin (2005)	US	1998-03	Veterans	Lab+symp	EIA Tox A	>72h/30day med hist	100	3.77 (3.1-4.59)	
NA	Koo (2014)	US	2010-11	≥18	Lab+symp	PCR,ELISA, Cul	All cases IP	NR	13.4 (11.8-15)	
NA	Koo (2014)	US	2010-11	≥18	Lab+symp	PCR,ELISA, Cul	All cases IP	NR	27 (24.7-29.3)	
NA	Mah (2011)	US	2007-08	≥20	Lab+symp	EIA Tox	during IP/IP<30 days	202	8.00 (6.97-9.18)	5.19 (4.52-5.96)
NA	Manian (2013)	US	2007-08	Adult	Lab+symp	EIA Tox A&B	>72 hours/IP 7d	322	8.80 (7.89-9.82)	
NA	McFarland (2007)	US	2004	Veterans	Lab+symp	Cul, EIA Tox A/&B	All cases	144	23.8 (20.21-28.02)	

Region	Reference	Country	Study yrs (if <1 year: mo/wk)	Age (years)	CDI ascertainment	Laboratory confirmation	HCF-CDI Incidence Rate (95% Confidence Interval)			
							Parameters	Cases	10,000 patient-days	1,000 admissions
NA	Murphy (2012)	US	2000-07	≥18	ICD9+History IP	No details	ICD9+PD<12 wks	5029		2.84 (2.77-2.92)
NA	Nowak (2012)	US	2003-06	Adult	Lab only	No details	All cases IP	1393	6.3 (5.98-6.64)	
NA	Salgado (2009)	US	2002-04	Adult	Lab+symp	EIA Tox A&B	>72 hours/IP 30 days	163	13.50 (11.58-15.74)	
NA	Tartof (2014)	US	2011-12	≥19	Lab	PCR	All cases IP	4784	35.42 (34.43-36.44)	
NA	Tartof (2015)	US	2011-12	≥18	Lab	PCR	All cases IP	2638	14.42 (13.88-14.98)	
NA	Welker (2012)	US	2006-07	>18	Lab+symp	EIA Tox A&B	>72 hours/IP 7 days	156		6.64 (5.67-7.76)
NA	Welker (2012)	US	2006-07	>18	ICD9	No details	>72 hours/IP 7 days	178		7.57 (6.54-8.77)
NA	Young-Xu (2015)	US	2009-13	≥18	ICD9+Lab+Tx	No details	During IP/IP<4ks	25976		13.26 (13.11-13.42)
NA	Zilberberg (2011)	US	2007-08	≥18	Lab	EIA Tox	>48 hours/IP<4 wks	6666	8.43 (8.23-8.64)	3.30 (3.22-3.38)
WP	Foster (2014)	Australia	2011-12 (6m)	Adult	Lab+symp	PCR	All cases	331	7.4 (6.64-8.24)	
WP	Honda (2014)	Japan	2010-12	≥18	Lab+symp	EIA ToxA&B, PCR	>72 hours/IP<4 wks	116	3.31 (2.76-3.97)	3.59 (2.99-4.31)
WP	Tan (2014)	Singapore	2011-12 (6m)	≥21	Lab+symp (i)	Tox A/B, GDH & PCR	>48 hours/IP<4 wks	47	7.62 (5.72-10.14)	4.54 (3.41-6.05)
WP	Kim (2013)	South Korea	2004-08	Adults	Lab+symp	EIA ToxA&/B, Cul	"healthcare-associated"	1890		2.74 (2.62-2.87)
WP	Chung (2010)	Taiwan	2007-08	≥18	Lab+symp	EIA Tox A&B	>48 hours/IP<4 wks	86	4.26 (3.45-5.26)	3.40 (2.75-4.20)

Notes: LA: Latin America, NA: North America, EU: Europe, WP: Western Pacific, EM: Eastern Mediterranean, AF: Africa. (i) incident cases only (i+r): incident and recurrent cases, IP: in-patient, ICD9: 008.45, LOS: Length of stay, EIA: Enzyme Immunoassay, Tox: Toxin, &: and, /: orCyt: Cytotoxin, GDH: glutamate dehydrogenase, Cul: Culture, PCR: polymerase chain reaction. Lab: Laboratory confirmation, Symp: symptoms confirmation, including at least one of the following diarrhoeal disease, endoscopic or histologic features.

Supplementary Table 3. HO-HCF CDI incidence density and number of cases per admissions (all ages and adults)

Region	Reference	Country	Study yrs (if <1 year: mo/wk)	Age (years)	CDI ascertainment	Laboratory assay	Healthcare onset-healthcare facility-CDI rates (95% Confidence Interval)			
							Parameters	Cases	10,000 patient-days	1,000 admissions
EU	ISP-WIV	Belgium	2008-14	All ages	Mandatory Surveillance (IP)	No details	>48 hours	1081	1.01 (0.95-1.07)	0.86 (0.81-0.91)
EU	Novak (2014)	Croatia	2010-11	All ages	Lab+symp (i)	EIA A&/B	facility-onset	39	0.45 (0.33-0.62)	0.37 (0.27-0.50)
EU	SSI (2015)	Denmark	2010-14	No details	Lab+symp	No details	≥48 hours	10397	5.98 (5.86-6.09)	
EU	Fenner (2008)	Switzerland	2006-07	All ages	Lab only (i)	EIA ToxA/B	≥2 days	75	2.21 (1.76-2.77)	2.55 (2.03-3.20)
LA	Souza Dias (2010)	Brazil	2002-03	All ages	Lab+symp	EIA Tox A&/B	>48 hours			3.32 (2.72-4.05)
NA	Alfa (2015)	Canada	2009-12	All wards	Lab	Multistep algorith	Healthcare-acquired	64	5.01 (3.92-6.4)	
NA	DiDiodato [08] (2013)	Canada	2008-11	≥1	MOHLTC	No details	>72 hours		3.20 (2.68-3.74)	
NA	Faires (2014)	Canada	2006-11	All ages	Lab+symp	EIA Tox A&B, GDH, PCR	>48 hours	86	1.12 (0.91-1.39)	
NA	Lambert (2009)	Canada	2005-06	All ages	Lab+symp	EIA GDH, Tox A, Cyt	>48 hours	515	7.27 (6.66-7.92)	
NA	Benoit (2011)	US	2007-08	All ages	Lab only	EIA	>72 hours	2156	7.82 (7.50-8.16)	
NA	Campbell (2009)	US	2006	No details	Lab only	EIA, Cyt, GDH, Cul	>48 hours	5700	7.11 (6.93-7.30)	
NA	Catanzaro (2012)	US	2009-10	No details	Lab+symp	EIA Tox A/B, PCR	≥48 hours	13	2.65 (1.53-4.6)	
NA	Dubberke (2006)	US	2003	No details	Lab+symp	EIA Tox A&B, ICD9	IP ≥2 days	662		14.55 (13.49-15.71)
NA	Durkin (2015)	US	2013	No details	Lab	No details	≥48 hours	311	4.4 (3.94-4.92)	
NA	Haas (2014)	US	2009-11	All ages	Lab	No details	>3 days	390	7.9 (7.16-8.72)	
NA	Hacek (2010)	US	2004-07	No details	Lab only	EIA Tox A/B	>48 hours		*8.5*	
NA	*Haley (2014)*	*US*	*2010*	*No details*	*LabID database*	*LabID*	*>72 hours*	*1608*	*10.99 (10.47-11.54)*	
NA	Haley (2014)	US	2010	No details	Audited	No details	>72 hours	1697	11.60 (11.06-12.17)	
NA	Han (2012)	US	2009 (6m)	No details	Lab only	EIA Tox A&B	>48 hours	346	15.45 (13.90-17.16)	
NA	Kassavin (2013)	US	2009 (9m)	All ages	Lab	GDH, EIA Tox A/B	>48 hours, no IP<4 wks	11		0.64 (0.35-1.15)
NA	Kazakova (2006)	US	2002-03	All ages	Lab	EIA Tox A	≥48 hours	9		1.9 (0.99-3.65)
NA	King (2011)	US	2009	No details	Lab+symp	ToxA&B	>48 hours	56		5.09 (3.91-6.61)
NA	Lipp (2012)	US	2007-08	All ages	ICD9+Present on admission code	No details	ICD9, no POA	1913		0.79 (0.75-0.82)
NA	McDonald (2012)	US	2010	No details	Lab	EIA ToxA/B, NAT, Other	Hospital-onset	9	7.4 (7.3-7.5)	
NA	McDonald (2012)	US	2008-10	No details	Lab+symp	No details	Hospital-onset	118	9.3 (8.7-9.8)	
NA	Muto (2007)	US	1996-99	No details	Lab+symp pre-outbreak	EIA, Cyt	≥72 hours	334	4.94 (4.43-5.49)	2.99 (2.69-3.33)
NA	Sohn (2005)	US	2000-03	No details	Lab+symp	Unknown	>48 hours	267	10.29 (9.13-11.60)	
NA	Tartof (2014)	US	2011-12	All ages	Lab only	PCR	>48 hours	952	6.78 (6.36-7.22)	3.54 (3.32-3.77)
WP	Mitchell (2011)	Australia	2006-10	≥ 2	Lab+symp	EIA, Cul	>48 hours	227	2.13 (1.87-2.43)	
WP	Mitchell (2014)	Australia	2007-10	≥ 2	Lab+symp	EIA ToxA&/B, PCR, Cul	≥48 hours	158	3.20 (2.74-3.74)	2.68 (2.28-3.13)
WP	Hikone (2015)	Japan	2011-13	≥ 2	Lab+symp	EIA	≥48 hours, IP<12 wks	76	0.8 (0.64-1)	
WP	Mori (2015)	Japan	2010-14	≥ 2	Lab+symp	PCR	>48 hours	35	1.6 (1.21-2.12)	
WP	Suzuki (2013)	Japan	2010-11	No details	Lab+symp	EIA	>48 hours	75	4.71 (3.76-5.91)	
WP	Gweon (2014)	Korea S	2011	All ages	Lab+symp	EIA Tox A/B, PCR	>48 hours	320	9.1 (8.15-10.16)	
	**Adults**									
AF	Rajabally (2013)	S. Africa	NR (1yr 4m)	≥18	Lab+symp	EIA Tox A, PCR	>48/LTCF <14d	40		0.87 (0.64-1.19)
EM	Khan (2012)	Qatar	2006-09	>15	Lab+symp	EIA ToxA&B	>72 hours	101	1.31 (1.08.1.60)	
EM	Al-Eidan (2013)	S. Arabia	2011	Adult	Lab only	ELISA ToxA&B	>48 hours	98	2.96 (2.43-3.61)	
EU	Khanafer (2015)	France	2011-13	>18	Lab+symp	EIA Tox A/B, Cul	>48 hours	118		4.22 (3.52-5.06)
EU	Vernaz (2009)	UK-Scotland	2004-08	>18		Infection control database	>48 hours	23		1.5 (1-2.26)
NA	Brown (2015)	Canada	2010-14	>18	Lab+symp	PCR	IP ≥2 days	255	5.95 (5.26-6.73)	
NA	Hamel (2010)	Canada	2001-06	>16	Lab+symp	EIA Tox A&B	>72 hours	202	8.09 (7.05-9.29)	5.36 (4.67-6.15)
NA	Oake (2010)	Canada	2002-09	≥15	Lab+symp	ToxA/B, Cul	IP ≥3 days	1393		10.18 (9.66-10.73)
NA	Dubberke [A] (2010)	US	2000-06	≥18	Lab	EIA Tox	>48 hours	2467	15.47 (14.88-16.10)	7.74 (7.44-8.05)
NA	Dubberke [B] (2010)	US	2000-06	≥18	Lab	EIA Tox	>48 hours	383	11.05 (10.00-12.21)	5.40 (4.88-5.97)
NA	Dubberke [C] (2010)	US	2000-06	≥18	Lab	EIA Tox	>48 hours	552	3.97 (3.65-4.32)	2.17 (2.00-2.36)
NA	Dubberke [D] (2010)	US	2000-06	≥18	Lab	EIA Tox	>48 hours	425	6.28 (5.71-6.91)	3.74 (3.40-4.11)
NA	Dubberke [E] (2010)	US	2000-06	≥18	Lab	EIA Tox	>48 hours	585	4.61 (4.25-5.00)	3.60 (3.32-3.91)

Region	Reference	Country	Study yrs (if <1 year: mo/wk)	Age (years)	CDI ascertainment	Laboratory assay	Healthcare onset-healthcare facility-CDI rates (95% Confidence Interval)			
							Parameters	Cases	10,000 patient-days	1,000 admissions
NA	Evans (2014)	US	2010-12	Veterans	Lab+symp (Aud, i+r)	EIA ToxA&B,PCR,Cyt,Cul	>48 hours	4439	8.40 (8.15-8.65)	
NA	*Evans (2014)*	*US*	*2010-12*	*Veterans*	*Lab (Lab)(i+r)*	*EIA ToxA&B,PCR,Cyt,Cul*	*>48 hours*	*4925*	*9.32 (9.06-9.58)*	
NA	Gase (2013)	US	2009 (6m)	≥18	Lab+symp (Aud, i+r)	EIA Tox A&B	>72 hours	1392	7.5 (7.12-7.91)	3.99 (3.79-4.21)
NA	*Gase (2013)*	*US*	*2009 (6m)*	*≥18*	*Lab only (LabID)*	*EIA Tox A&B*	*>72 hours*	*1793*	*9.66 (9.22-10.11)*	*5.13 (4.90-5.38)*
NA	Howell (2010)	US	2004-08	≥18	Lab only	Tox +	IP≥3 days	665		6.53 (6.05-7.05)
NA	Jiang (2013)	US	2010-11	≥18	ICD9	No details	no POA, ICD-9	1211		5.36 (5.06-5.67)
NA	Knight (2010)	US	2001-03	≥18	Lab only	EIA Tox A&B	>72 hours	270	4.96 (4.4-5.59)	
NA	Murphy (2012)	US	2000-07	≥18	ICD9+History IP	No details	no POA	1952		1.10 (1.06-1.15)
NA	Price (2007)	US	2002-04	No details	Lab+symp	Cyt, EIA Tox B	>48 hours	213	7.00 (6.12-8.01)	
NA	Schmiedeskamp (2009)	US	2004-05	≥18	Lab+symp (Aud)	EIA Tox A&B	>5 days	62		2.59 (2.02-3.32)
NA	Song (2008)	US	2000-05	≥18	Lab+symp	EIA Tox A&B,Cyt,Cul	≥72 hours	741	7.20 (6.70-7.74)	
NA	Zilberberg (2011)	US	2007-08	≥18	Lab only (LabID)	EIA Tox	>48 hours	4963	6.28 (6.1-6.45)	2.45 (2.39-2.52)
NA	Young-Xu (2015)	US	2009-13	≥18	ICD9+Lab+Tx	No details	During IP	20046	6.94 (6.84-7.04)	10.23 (10.09-10.38)
WP	Mitchell (2014)	Australia	2007-10	≥20	Lab+symp	Tox, PCR A&/B, Cul	IP≥2 days	149		2.77 (2.36-3.25)
WP	Honda (2014)	Japan	2010-12	≥18	Lab+symp	EIA ToxA&B, PCR	>72 hours	109	3.11 (2.58-3.76)	3.38 (2.80-4.07)
WP	Han (2014)	S. Korea	2011-12	≥20	Lab+symp	EIA ToxA&/B, Cul	>48 hours	98	2.7 (2.22-3.29)	
WP	Tan (2014)	Singapore	2011-12 (6m)	≥21	Lab+symp (i)	Tox A/B, GDH & PCR	>48 hours	33	5.35 (3.80-7.53)	3.19 (2.27-4.49)

Notes: LA: Latin America, NA: North America, EU: Europe, WP: Western Pacific, EM: Eastern Mediterranean, AF: Africa. (i) incident cases only (i+r): incident and recurrent cases, IP: in-patient, ICD9: 008.45, EIA: Enzyme Immunoassay, Tox: Toxin, &: and, /: or Cyt: Cytotoxin, GDH: glutamate dehydrogenase, Cul: Culture, PCR: polymerase chain reaction. Lab: Laboratory confirmation, Symp: symptoms confirmation, including at least one of the following diarrhoeal disease, endoscopic or histologic features.

Supplementary Table 4. Number of CA-CDI cases per admissions and cumulative incidence rates, and cumulative incidence rates of all CDI (all ages and adults)

Region	Reference	Country	Study years	Age (years)	CDI ascertainment	Laboratory assay	CA-CDI Incidence Rate (95% Confidence Interval)			Any CDI Incidence Rate (95% Confidence Interval)	
							Parameters	N	1,000 admission		
EU	Novak (2014)	Croatia	2010-11	All ages	Lab+symp (i)	EIA ToxA/B	No IP>12 wks	12	0.11 (0.06-0.20)		
EU	Barbut (2007)	France	2000-04	All ages	Lab only	Cyt,Cul,EIA,PCR	No IP>4 wks, <72hours IP	28	0.20 (0.14-0.30)		
EU	Eckert (2013)	France	2009	No details	Lab+symp	EIA,Cyt,PCR,Cul,GDH	No IP>12 wks	369	0.31 (0.28-0.34)		
EU	Jen (2012)	UK-England	1997-10	≥2	ICD-10+no IP in 90d	No details	No IP>12 wks or LOS <72 hours	21221	0.24 (0.24-0.24)		
LA	Souza Dias (2010)	Brazil	2002-03	All ages	Lab+symp	Cul,PCR	within 48 hours IP	21	0.71 (0.46-1.09)		
NA	King (2011)	US	2009	All ages	Lab+symp	ToxA&B	No IP>12 wks	26	2.36 (1.61-3.47)		
NA	Tartof (2014)	US	2011-12	≥1	Lab only	PCR	No IP>12 wks	1502	5.59 (5.32-5.88)		
	Adults										
EU	Jen (2012)	UK-England	1997-10	≥2	ICD-10+no IP in 90d	No details	No IP>12 wks or <72hours IP	20884	0.26 (0.26-0.26)		
NA	Armbuster (2012)	US	2005-09	Army staff	Lab only	No details	No IP>12 wks	68	0.97 (0.76-1.23)		
NA	Bengualid (2011)	US	2006-08	≥18	Lab +symp	EIA ToxA/B	No IP>6 months, and <48 hours	22	0.43 (0.29-0.66)		
NA	Dubberke [A] (2010)	US	2000-06	≥18	Lab only	EIA Tox	No IP>12 wks, <48 hours	203	0.64 (0.55-0.73)		
NA	Dubberke [B] (2010)	US	2000-06	≥18	Lab only	EIA Tox	No IP>12 wks, <48 hours	67	0.94 (0.74-1.20)		
NA	Dubberke [C] (2010)	US	2000-06	≥18	Lab only	EIA Tox	No IP>12 wks, <48 hours	21	0.08 (0.05-0.13)		
NA	Dubberke [D] (2010)	US	2000-06	≥18	Lab only	EIA Tox	No IP>12 wks, <48 hours	78	0.69 (0.55-0.86)		
NA	Dubberke [E] (2010)	US	2000-06	≥18	Lab only	EIA Tox	No IP>12 wks, <48 hours	53	0.33 (0.25-0.43)		
NA	Evans (2014)	US	2010-12	Veterans	Lab+symp	Tox, Cul	LabID	3363	0.35 (0.34-0.36)		
NA	Murphy (2012)	US	2000-07	Adult	ICD9+PD	No details	1st code, no IP>90 days	5667	3.20 (3.12-3.29)		
NA	Zilberberg (2011)	US	2007-08	≥18	LabID+ Chart review	EIA Tox	No IP>12 wks, <48 hours	2254	1.11 (1.07-1.16)		
WP	Honda (2014)	Japan	2010-12	≥18	Lab+symp	EIA,PCR	No IP>12 wks	4	0.12 (0.05-0.33)		
							**Parameters**	**N**	**100,000 population**	**N**	**100,000 population**
EU	Soes (2014)	Denmark	2009-11	≥2	Lab+symp	Tox, Cul	No IP>12 wks, <48 hours	98	22.43 (18.4-27.34)		
EU	Hensgens (2014)	Netherlands	2010-12	≥2	Lab+symp	Tox, Cyt	GP-identified	194	6.70 (5.82-7.71)		
EU	Reigadas (2015)	Spain	2013	≥2	Lab+symp	Tox	No IP>12 wks, <72 hours	76	47.8 (38.18-59.85)		
EU	Fellmeth (2010)	UK-Eng	2008-09	2-64	Lab only	EIA Tox A&B	Community onset	54	12.92 (9.89-16.87)		
EU	Wilcox (2008)	UK-Eng Leeds	1999	All ages	Lab+symp	Cul,Cyt	GP-identified	21	29.50 (19.23-45.25)		
EU	Wilcox (2008)	UK-Eng Truro	1999	All ages	Lab+symp	Cul,Cyt	GP-identified	21	20.20 (13.17-30.98)		
EU	Taori (2014)	UK-Scotland	2010-11	All ages	Lab	Two-step alg	No HCF >12wks	42	6.4 (4.73-8.66)	335	50.39 (45.28-56.09)
NA	Lambert (2009)	Canada	2005-06	All ages	Lab	Tox	No IP>12 wks	275	23.40 (20.79-26.34)	1006	85.60 (80.47-91.06)
NA	CDC (2005)	US	2004-05	No details	Voluntary Survey	Tox,Cul	No IP>12 wks, <72 hours	276	7.60 (6.75-8.55)		
NA	CDC (2008)	US	2006	All ages	Lab+symp	EIA	No IP>12 wks, <72 hours	241	6.90 (6.08-7.83)		
NA	Chandler (2007)	US	1995-07 (95,02 IR)	No details	Lab only	No details				1686	24.5 (22.9-26.22)
NA	Khanna (2012)	US	1991-05	All ages	ICD9 (OP/IP) chart review	(Cyt,EIA)	No IP>12 wks, <48 hours	157	9.60 (8.21-11.23)	385	25.2 (22.80-27.85)
NA	Kuntz (2011)	US	2004-07	All ages	ICD9 (OP/IP)	No details	1st code, IP<12 wks	304	11.16 (9.98-12.49)	684	25.12 (23.3-27.08)
NA	Lessa (2015)	US	2011	≥1	Lab	Tox,PCR	No IP>12 wks	159700	51.9 (43.2-60.5)	453000	147.2 (129.1-165.3)
NA	Kuntz (2015)	USA	2005-12	All ages	ICD-9+Tx	GDH, Tox A/B, PCR	OP, No IP>12 wks			5990	159 (154.97-163.13)
WP	Mitchell (2012)	Australia	2010-11	>2	Lab+symp	EIA,Cul ToxA/B,PCR	No IP>12 wks	71	6.98 (5.02-9.7)	259	45.11 (39.64-51.34)

Region	Reference	Country	Study years	Age (years)	CDI ascertainment	Laboratory assay	CA-CDI Incidence Rate (95% Confidence Interval)			Any CDI Incidence Rate (95% Confidence Interval)	
							Parameters	N	1,000 admission		
EU	Alcalá (2011)	Spain	2007	No details	Lab only	EIA, Cyt, PCR (ToxB), Cul				4123	13.42 (13.02-13.84)
NA	Babey	Canada	2012-14	≥2	Lab+symp	Tox				34	24.29 (17.35-33.99)
EU	ISP-WIV	Belgium	1999-12	All ages	ICD-9					3472	32.7 (31.63-33.81)
EU	HPSC (2013)	Ireland	2013	All ages	Lab+symp	EIA+GDH&PCR				1853	39.38 (37.61-41.23)
EU	NIHW	Finland	2008-12	No details	Mandatory Surv (IP)	Tox+				5489	103.05 (100.34-105.83)
EU	Lyytikainen (2009)	Finland	1996-04	All ages	ICD-9					10958	23.6 (22.31-24.97)
EU	PHE	UK England	2007-12	≥2	Lab+symp M-Surv	EIA+GDH				199146	47.15 (46.55-47.76)
EU	Rodriguez-Pardo (2013)	Spain	2009	All ages	Lab+symp	Tox A&B, Cul				365	22.50 (20.31-24.93)
EU	Folkhälsomyndigheten	Sweden	2014	All ages	Voluntary surv programme					7691	79 (77.22-80.82)
EU	Vesteinsdottir (2012)	Iceland	2010-11	All ages	Lab only	ELISA Tox A&B				146	45.85 (38.99-53.92)
WP	Ferguson (2011)	Australia	2008-09	All ages	Lab Survey	Various: Cul, EIA A&B, GDH, PCR (6%)				944	21.9 (20.5-23.3)
WP	Ferguson (2011)	New Zealand	2008-09	All ages	Lab Survey	Various: Cul, EIA A&B, GDH, PCR (6%)				5625	25.6 (24.9-26.3)
NA	Smith (2011)	US	1999-08	All ages	ICD9 008.45	No details				19174	43.82 (41.89-45.84)
NA	VerLee (2012)	US	2002-08	All ages	ICD9 008.45	No details				68686	98.7 (98.0-99.5)
NA	Lucado (2012)	US	1993-09	All ages	ICD9 008.45	No details				336471	107.6 (107.3-108.0)
	Adults										
NA	CDC (2008)	US	2006	Adults	Voluntary Survey	EIA	No IP>12 wks, <72 hours	223	7.94 (6.97-9.06)		
NA	Gutierrez (2013)	US	1998-10	Army staff	ICD9+history admissions review	No details	No IP>12w	1018	5.50 (5.17-5.85)	2423	13.2 (12.7-13.7)
NA	Murphy (2012)	USA	2000-07	≥18	ICD9+history admissions review	No details	No IP>12w	5667	14.3 (13.94-14.67)		
NA	Kutty (2010)	US	2005	≥18	Lab+symp	EIA Tox A&B	No IP>8 wks, OP/3-4 days IP	73	46.00 (36.57-57.86)		
NA	Lessa (2015)	US	2011	≥18	Lab+symp	PCR, Tox	No IP>12 wks or <72hours IP	147200	61.88 (61.57-62.19)	436100	183.41 (182.87-183.96)
EU	Dial (2005)	UK	1994-04	≥18	Lab/symp	No details	GP-identified in database	1233	8.64 (8.17-9.14)		
EU	Soes (2014)	Denmark	2009-11	Adults	Lab+symp	Tox, Cul	No IP>12 wks or <72hours IP	92	27.08 (22.07-33.23)		
NA	Young-Xu (2015)	USA	2009-13	≥18	ICD9+Lab+Tx	No details	No IP >12wks	5168	91.72 (89.23-94.29)		
EU	Taori (2014)	UK-Scotland	2010-11	Adults	Lab	Two-step alg	No HCF >12wks		7.3 (NR)		
EU	Vesteinsdottir (2012)	Iceland	2010-11	>18	Lab only	ELISA Tox A&B	No IP>6 wks, no nursing home history	30	12.60 (8.81-18.02)	128	53.77 (45.22-63.93)
NA	Kuntz (2012)	US	2007	≥20	ICD9+Lab	EIA Tox				870	149 (139.42-159.24)
NA	Rhee (2014)	US	2012	>1	Lab	EIA Tox, GDH				1666	285.03 (276.73-293.58)
EU	Kotila (2011)	Finland	2008	≥15	Mandatory & Enhanced-Surv (IP)	Cul, Tox				6201	137.1 (133.76-140.53)
EU	PHE	UK-England	2006-14	Adults	Lab					26980	62.01 (61.28-62.75)
WP	Mitchell (2012)	Australia	2010-11	≥20	Lab+symp	Cul,EIA Tox,PCR				444	58.93 (51.65-67.24)
NA	Lucado (2012)	US	1993-09	≥20	ICD9 008.45	No details				328053	137.8 (137.3-138.3)
NA	Smith (2011)	US	1999-08	≥15	ICD9 008.45	No details				18503	54.28 (53.51-55.07)
NA	VerLee (2012)	US	2002-08	≥20	ICD9 008.45	No details				8834	16.23 (15.36-17.16)
NA	Zilberberg (2008)	US	2000-05	≥18	ICD9 008.45	No details				1200343	92.43 (92.26-92.59)

Region	Reference	Country	Study years	Age (years)	CDI ascertainment	Laboratory assay	CA-CDI Incidence Rate (95% Confidence Interval)			Any CDI Incidence Rate (95% Confidence Interval)
							Parameters	N	1,000 admission	

Notes: LA: Latin America, NA: North America, EU: Europe, WP: Western Pacific, EM: Eastern Mediterranean, AF: Africa. (i) incident cases only (i+r): incident and recurrent cases, IP: in-patient, ICD9: 008.45, ICD10: A047, EIA: Enzyme Immunoassay, Tox: Toxin, &: and, /: orCyt: Cytotoxin, GDH: glutamate dehydrogenase, Cul: Culture, PCR: polymerase chain reaction. Lab: Laboratory confirmation, Symp: symptoms confirmation, including at least one of the following diarrhoeal disease, endoscopic or histologic features, or unformed stool. Surv: Surveillance, LOS: Length of stay

Supplementary Table 5. Number of any CDI cases per admissions (all ages and adults)

Region	Reference	Country	Study yrs (if <1 year: months /wks)	Age (years)	CDI ascertainment	Laboratory confirmation	All-CDI Incidence Rate (95% Confidence Interval)	
							Cases	1,000 admissions
EM	Al-Tawfiq (2010)	S. Arabia	2007-08	All ages	Lab+symp	ELISA Tox A&B	42	1.05 (0.78-1.42)
EU	ISP-WIV	Belgium	2008-14	≥0	M-Surv (IP)	No details	14672	1.62 (1.59-1.64)
EU	Ivanova (2011)	Bulgaria	2008-10	> 2	Lab+symp (i)	EIA ToxA&B, Cul	15	0.79 (0.48-1.31)
EU	Novak (2014)	Croatia	2010-11	All ages	Lab+symp (i)	EIA A&/B	54	0.51 (0.39-0.67)
EU	Barbut (2007)	France	2000-04	All ages	Lab+symp	Cyt, Cul, EIA, PCR	151	1.10 (0.94-1.29)
EU	Eckert (2013)	France	2009 (6m)	No details	Voluntary Survey Lab+symp (i)	EIA Tox, Cyt, PCR, Cul, GDH	1316	1.10 (1.04-1.16)
EU	Jones (2012)	France	2000-10	No details	Lab	Cyt, Cul	632	1.99 (1.84-2.16)
EU	KISS (2015)	Germany	2007-14	All ages	V-Surv	No details	95545	4.83 (4.8-4.86)
EU	Hensgens (2013)	Netherlands	2006-09	All ages	Lab+symp (i)	ELISA Tox A&/B, Cyt	1366	1.33 (1.26-1.40)
EU	Kooi [01] (2008)	Netherlands	2004-05	No details	Lab only	Tox	34	1.71 (1.22-2.39)
EU	Kooi [02] (2008)	Netherlands	2004-05	No details	Lab only	Tox	58	1.3 (1.01-1.68)
EU	Kooi [03] (2008)	Netherlands	2004-05	No details	Lab only	Tox	136	3.59 (3.04-4.25)
EU	Kooi [04] (2008)	Netherlands	2004-05	No details	Lab only	Tox	50	2.6 (1.97-3.43)
EU	Kooi [05] (2008)	Netherlands	2004-05	No details	Lab only	Tox	83	3.36 (2.71-4.17)
EU	Kooi [06] (2008)	Netherlands	2004-05	No details	Lab only	Tox	29	1.47 (1.02-2.12)
EU	Kooi [07] (2008)	Netherlands	2004-05	No details	Lab only	Tox	24	0.59 (0.4-0.88)
EU	Kooi [08] (2008)	Netherlands	2004-05	No details	Lab only	Tox	21	0.49 (0.32-0.75)
EU	Kooi [09] (2008)	Netherlands	2004-05	No details	Lab only	Tox	65	1.2 (0.94-1.53)
EU	Kooi [10] (2008)	Netherlands	2004-05	No details	Lab only	Tox	94	3.01 (2.46-3.68)
EU	Kooi [11] (2008)	Netherlands	2004-05	No details	Lab only	Tox	34	1.03 (0.74-1.44)
EU	Kooi [12] (2008)	Netherlands	2004-05	No details	Lab only	Tox	30	1.16 (0.81-1.66)
EU	Kooi [13] (2008)	Netherlands	2004-05	No details	Lab only	Tox	109	3.79 (3.14-4.58)
EU	Kooi [14] (2008)	Netherlands	2004-05	No details	Lab only	Tox	19	0.81 (0.52-1.27)
EU	Kooi [15] (2008)	Netherlands	2004-05	No details	Lab only	Tox	20	1.07 (0.69-1.66)
EU	Kooi [16] (2008)	Netherlands	2004-05	No details	Lab only	Tox	1	0.06 (0.01-0.43)
EU	Kooi [17] (2008)	Netherlands	2004-05	No details	Lab only	Tox	33	1.05 (0.75-1.48)
EU	Kooi [18] (2008)	Netherlands	2004-05	No details	Lab only	Tox	65	1.97 (1.55-2.51)
EU	Kooi [19] (2008)	Netherlands	2004-05	No details	Lab only	Tox	9	0.52 (0.27-1)
EU	Kooi [20] (2008)	Netherlands	2004-05	No details	Lab only	Tox	60	1.35 (1.05-1.74)
EU	Kooi [21] (2008)	Netherlands	2004-05	No details	Lab only	Tox	44	4.9 (3.65-6.59)
EU	Kooi [22] (2008)	Netherlands	2004-05	No details	Lab only	Tox	16	0.38 (0.23-0.62)
EU	Kooi [23] (2008)	Netherlands	2004-05	No details	Lab only	Tox	30	0.69 (0.48-0.99)
EU	Kooi [24] (2008)	Netherlands	2004-05	No details	Lab only	Tox	44	1.07 (0.8-1.44)
EU	Paltansing [1] (2007)	Netherlands	2005	All ages	Lab+symp (i)	Tox A&B EIA	11	4.60 (2.55-8.31)
EU	Paltansing [2] (2007)	Netherlands	2005	All ages	Lab+symp (i)	Tox A&B EIA	13	3.70 (2.15-6.37)
EU	Paltansing [3] (2007)	Netherlands	2005	All ages	Lab+symp (i)	Tox A&B Cyt	3	1.60 (0.52-4.96)
EU	Paltansing [4] (2007)	Netherlands	2005	All ages	Lab+symp (i)	Tox A&B Cyt	6	1.70 (0.76-3.78)
EU	Paltansing [5] (2007)	Netherlands	2005	All ages	Lab+symp (i)	Tox A&B EIA	4	1.40 (0.53-3.73)
EU	Paltansing [6] (2007)	Netherlands	2005	All ages	Lab+symp (i)	Tox A&B Cyt	7	1.30 (0.62-2.73)
EU	Paltansing [7] (2007)	Netherlands	2005	All ages	Lab+symp (i)	Tox A&B Cyt	3	1.40 (0.45-4.34)
EU	Paltansing [8] (2007)	Netherlands	2005	All ages	Lab+symp (i)	Tox A&B EIA	1	0.30 (0.04-2.13)
EU	Paltansing [9] (2007)	Netherlands	2005	All ages	Lab+symp (i)	Tox A&B EIA + Cyt	4	0.20 (0.08-0.53)
EU	Paltansing [10] (2007)	Netherlands	2005	All ages	Lab+symp (i)	Tox A&B Cyt	3	0.40 (0.13-1.24)
EU	Paltansing [11] (2007)	Netherlands	2005	All ages	Lab+symp (i)	Tox A&B Cyt	2	0.50 (0.13-2.00)
EU	Paltansing [12] (2007)	Netherlands	2005	All ages	Lab+symp (i)	Tox A&B Cyt	6	1.40 (0.63-3.12)
EU	Paltansing [13] (2007)	Netherlands	2005	All ages	Lab+symp (i)	Tox A&B EIA	4	1.20 (0.45-3.20)
EU	Czepiel (2015)	Poland	2008-14	No details	Lab+symp	Antigen and Tox A/B	1009	2.87 (2.7-3.05)
EU	Alcalá (2011)	Spain	2007	No details	Lab only	EIA, Cyt, PCR (ToxB), Cul	4123	1.71 (1.66-1.76)
EU	Rodriguez-Pardo (2013)	Spain	2009	All ages	Lab+symp	Tox A&B, Cul	365	1.22 (1.10-1.35)

Region	Reference	Country	Study yrs (if <1 year: months /wks)	Age (years)	CDI ascertainment	Laboratory confirmation	All-CDI Incidence Rate (95% Confidence Interval)	
							Cases	1,000 admissions
EU	Rodriguez-Pardo (2013)	Spain	2009	All ages	Lab+symp	Tox A&B, Cul	125	2.61 (2.19-3.11)
EU	Rodriguez-Pardo (2013)	Spain	2009	All ages	Lab+symp	Tox A&B, Cul	46	1.92 (1.44-2.57)
EU	Rodriguez-Pardo (2013)	Spain	2009	All ages	Lab+symp	Tox A&B, Cul	53	1.1 (0.84-1.44)
EU	Rodriguez-Pardo (2013)	Spain	2009	All ages	Lab+symp	Tox A&B, Cul	72	2.12 (1.68-2.67)
EU	Rodriguez-Pardo (2013)	Spain	2009	All ages	Lab+symp	Tox A&B, Cul	3	0.24 (0.08-0.75)
EU	Rodriguez-Pardo (2013)	Spain	2009	All ages	Lab+symp	Tox A&B, Cul	3	0.15 (0.05-0.45)
EU	Rodriguez-Pardo (2013)	Spain	2009	All ages	Lab+symp	Tox A&B, Cul	11	5.97 (3.31-10.79)
EU	Rodriguez-Pardo (2013)	Spain	2009	All ages	Lab+symp	Tox A&B, Cul	13	0.82 (0.48-1.42)
EU	Rodriguez-Pardo (2013)	Spain	2009	All ages	Lab+symp	Tox A&B, Cul	4	0.21 (0.08-0.56)
EU	Rodriguez-Pardo (2013)	Spain	2009	All ages	Lab+symp	Tox A&B, Cul	5	0.33 (0.14-0.79)
EU	Rodriguez-Pardo (2013)	Spain	2009	All ages	Lab+symp	Tox A&B, Cul	5	0.74 (0.31-1.79)
EU	Rodriguez-Pardo (2013)	Spain	2009	All ages	Lab+symp	Tox A&B, Cul	15	0.71 (0.43-1.17)
EU	Rodriguez-Pardo (2013)	Spain	2009	All ages	Lab+symp	Tox A&B, Cul	2	0.19 (0.05-0.75)
EU	Rodriguez-Pardo (2013)	Spain	2009	All ages	Lab+symp	Tox A&B, Cul	0.05000	0 (0-30.62)
EU	Rodriguez-Pardo (2013)	Spain	2009	All ages	Lab+symp	Tox A&B, Cul	1	0.16 (0.02-1.12)
EU	Fenner (2008)	Switzerland	2006-07	All ages	Lab only (i)	EIA ToxA/B	78	2.65 (2.12-3.31)
EU	Aldeyab (2009)	UK-N.IRE	2002-07	≥2	Lab+symp	Tox A&B	393	1.93 (1.75-2.13)
LA	Goorhuis (2009)	Argentina	2000-05	No details	Lab+symp	Cul, EIA Tox, PCR	153	5.45 (4.65-6.39)
LA	Souza Dias (2010)	Brazil	2002-03	All ages	Lab+symp	EIA A&/B	138	4.74 (4.01-5.61)
LA	Camacho-O (2009)	Mexico	2003-07	No details	Lab+symp	EIA Tox	170	5.04 (4.34-5.86)
NA	Catanzaro (2012)	US	2009-10	No details	Lab only	EIA, Cyt, GDH, Cul	57	4.94 (3.82-6.41)
NA	Kassavin (2013)	US	2009 (9m)	All ages	Lab+clinical suspicion	GDH, EIA Tox A/B	88	5.10 (4.14-6.29)
NA	King (2011)	US	2009	No details	Lab+symp	ToxA&B	115	10.45 (8.70-12.54)
NA	Tan (2007)	US	2000-04	All ages	Lab+symp	Tox	13394	5.66 (5.57-5.76)
NA	Tartof (2014)	US	2011-12	All ages	Lab only	PCR	4880	18.16 (17.66-18.68)
EU	Jones (2012)	France	2000-10	No details	ICD 10	NA	284	0.90 (0.80-1.01)
EU	Stausberg (2011)	Germany	2006	All ages	ICD-9		36215	2.39 (2.37-2.41)
NA	Daneman (2012)	Canada	2002-08	All ages	ICD10	No details	33634	5.54 (5.48-5.60)
NA	Gilca (2010)	Canada	1998-01	No details	ICD9	No details	10304	5.80 (5.69-5.92)
NA	Chandler (2007)	US	1995-07	No details	Lab only	No details	442	1.08 (1.07-1.09)
NA	Elixhauser (2008)	US	1993-05	No details	ICD9	No details	2773521	7.69 (7.6-7.72)
NA	Fashner (2012)	US	2000-09	All ages	ICD-9		72	5.38 (4.27-6.78)
NA	Halabi (2013)	US	2001-10	All ages	ICD9	No details	301200	6.82 (6.82-6.83)
NA	Harbrecht (2012)	US	2003-05	All ages	ICD9	No details		4.92 (4.8-5.0)
NA	O'Brien (2007)	US	2000	All ages	ICD-9		1036	9.35 (8.8-9.94)
NA	Ricciardi (2007)	US	1993-03	All ages	ICD9	No details	299453	3.83 (3.82-3.85)
NA	Ricciardi (2008)	US	2000-03	All ages	ICD9	No details	80619	3.97 (3.95-4.00)
WP	Cheng (2015)	Hong Kong	2009	All ages	Lab	Cul, Cyt	307	0.95 (0.85-1.06)
WP	Chan (2011)	Singapore	2007	All ages	ICD9		141	2.50 (2.12-2.95)
WP	Chan (2011)	Singapore	2007	All ages	Lab	ELISA Tox A&B	276	4.90 (4.35-5.51)
WP	Koh (2007)	Singapore	2002-03 (5m)	All ages	Lab+symp (IP)	EIA A&B, Cul	88	3.20 (2.60-3.94)
	Adults							
AF	Rajabally (2013)	S. Africa	NR (1yr 4m)	≥18	Lab+symp	EIA Tox A, PCR	59	1.28 (0.99-1.66)
EU	Balihar (2014)	Czech Rep	2006-08	≥18	Lab+symp	ELISA, EIA ToxA/B	183	0.97 (0.84-1.13)
EU	Skyum (2014)	Denmark	2012-13	≥16	Lab+symp	PCR, Cul	38	2.17 (1.58-2.98)
EU	Drudy (2007)	Ireland	2004 (6m)	Adult	Lab+symp	ELISA Tox A&B, Cyt	85	7.0 (5.66-8.66)
EU	Laza (2015)	Romania	2013-14	≥18	Lab+symp	EIA Tox A&B	219	19.65 (17.21-22.44)
NA	Armbruster (2012)	US	2005-09	Army staff	Lab only	No details	232	3.30 (2.90-3.75)
NA	Bengualid (2011)	US	2006-08	≥18	Lab+symp	EIA Tox A&B	352	6.90 (6.22-7.66)
NA	Dubberke [A] (2010)	US	2000-06	≥18	Lab only	EIA Tox	3928	12.32 (11.94-12.71)

Region	Reference	Country	Study yrs (if <1 year: months /wks)	Age (years)	CDI ascertainment	Laboratory confirmation	All-CDI Incidence Rate (95% Confidence Interval)	
							Cases	1,000 admissions
NA	Dubberke [B] (2010)	US	2000-06	≥18	Lab only	EIA Tox	541	7.62 (7.01-8.30)
NA	Dubberke [C] (2010)	US	2000-06	≥18	Lab only	EIA Tox	882	3.47 (3.25-3.71)
NA	Dubberke [D] (2010)	US	2000-06	≥18	Lab only	EIA Tox	621	5.47 (5.05-5.91)
NA	Dubberke [E] (2010)	US	2000-06	≥18	Lab only	EIA Tox	934	5.75 (5.40-6.13)
NA	Evans (2014)	US	2010-12	Veterans	Lab+symp (Audited, i+r)	EIA Tox A&B,PCR,Cyt,Cul	9642	10.06 (9.86-10.26)
NA	Gase (2013)	US	2009 (6m)	≥18	Lab+symp (Audited, i+r)	EIA Tox A&B	3301	9.45 (9.13-9.78)
NA	Jayatilaka (2007)	US	2001-05	≥18	ICD9	No details	519	6.75 (6.19-7.36)
NA	Jiang (2013)	US	2010-2011	≥18	ICD9	No details	4531	20.05 (19.47-20.64)
NA	Murphy (2012)	US	2000-07	≥18	ICD9	No details	10750	6.08 (5.96-6.19)
NA	Reveles (2014)	US	2001-10	≥18	ICD9	No details	2196446	6.91 (6.90-6.92)
NA	Salazar (2009)	US	2005-07	≥18	Lab+symp	EIA Tox	467	3.28 (3.00-3.59)
NA	Schmiedeskamp (2009)	US	2004-05	≥18	Lab+symp (Audited)	EIA Tox A&B	118	4.93 (4.12-5.91)
NA	Schmiedeskamp (2009)	US	2004-05	≥18	ICD9	No details	147	6.15 (5.23-7.22)
NA	Tartof (2014)	US	2011-12	≥19	Lab only	PCR	4880	18.16 (17.66-18.68)
NA	Welker (2012)	US	2006-07	>18	Lab only+symp (Audited)	EIA Tox A&B	384	16.30 (14.75-18.01)
NA	Welker (2012)	US	2006-07	>18	ICD9	No details	446	18.98 (17.30-20.83)
NA	Zilberberg (2011)	US	2007-08	≥18	LabID database	EIA Tox	9803	4.85 (4.75-4.94)
NA	Chopra (2015)	US	2012	Adults	ICD-9	No details	615	11.98 (11.07-12.96)
NA	Cooper (2013)	US	2008-11	Adults	Lab only	Toxin positive	488	11.03 (10.09-12.06)
NA	Young-Xu (2015)	US	2009-13	≥18	ICD-9-IP, Lab, Tx records	No details	32593	16.64 (16.46-16.82)
WP	Honda (2014)	Japan	2010-12	≥18	Lab+symp	EIA ToxA&B, PCR	126	3.90 (3.28-4.65)
WP	Koh (2007)	Singapore	2002-03 (5m)	≥20	Lab+symp (IP)	EIA A&B, Cul	85	3.22 (2.60-3.98)
WP	Tan (2014)	Singapore	2011-12 (6m)	≥21	Lab+symp (i)	Tox A/B, GDH & PCR	66	6.38 (5.01-8.12)

Notes: LA: Latin America, NA: North America, EU: Europe, WP: Western Pacific, EM: Eastern Mediterranean, AF: Africa. (i) incident cases only (i+r): incident and recurrent cases, IP: in-patient, ICD9: 008.45, EIA: Enzyme Immunoassay, Tox: Toxin, &: and, /: orCyt: Cytotoxin, GDH: glutamate dehydrogenase, Cul: Culture, PCR: polymerase chain reaction, LAMP: loop-mediated isothermal amplification, Lab: Laboratory confirmation, Symp: symptoms confirmation, including at least one of the following diarrhoeal disease, endoscopic or histologic features.

Supplementary Table 6. CDI incidence in selected high risk settings, long-term care facilities (LTCF), intensive care units (ICU) and internal medicine (Intern Med)

Author	Country	Setting	Study Years	Parameters	Case ascertainment	Laboratory assay	Cases	CDI incidence rates and 95% confidence intervals	
								10,000 pt-days	1,000 admission
Brakovich (2013)	US	LTCF	2006-09	≥ 3 days (pre-intervention)	Lab+symp	No details	170	46.86 (40.32-54.46)	
Campbell (2009)	US	LTCF (i)	2006	≥ 2 days incident cases	Lab	EIA	6900	2.31 (2.25-2.36)	
Kim (2011)	US	LTCF	2004-10	≥ 4-30 days	ICD9+chart	Lab positive charge,Tx	96	1.67 (1.37-2.04)	
Laffan (2006)	US	LTCF	2001-03	≥ 3 days	Lab+symp	EIA	119	6.23 (5.21-7.46)	
Hooker (2015) [A]	USA	LTCF	2012-13	Onset in ward	Lab	NAAT	35	11.77 (8.45-16.4)	
Hooker (2015) [B]	USA	LTCF	2012-13	Onset in ward	Lab	NAAT	11	12.99 (7.19-23.46)	
Kazakova (2006)	USA	LTCF	2002-03	Onset in ward	Lab+symp	Tox A	2		0.4 (0.1-1.6)
Zarowitz (2015)	USA	LTCF	2009-10	Onset in ward	ICD9+Tx	No details	2048	0.98 (0.94-1.02)	
Eckert (2013)	France	LTCF	2009 (6m)	≥ 2 days	Lab+symp	Various	286	0.94 (0.84-1.06)	
Kim (2011)	US	LTCF	2004-10	≥ 30 days	Lab+symp	Lab positive charge,Tx	66	1.65 (1.30-2.10)	
Mylotte (2013)	US	LTCF	2009-11	≥ 30 days	Lab+symp	EIA	12	0.56 (0.32-0.99)	
McFarland (2007)	US	LTCF	2004	All cases	Lab+symp	EIA	17	4.20 (2.61-6.76)	
Pawar (2012)	US	LTCF (i)	2010	All cases incident cases	Lab+symp	EIA	425	2.26 (2.06-2.49)	
Laffan (2006)	US	LTCF	2001-03	All cases	Lab+symp	EIA	186	9.74 (8.44-11.3)	
Eckert (2013)	France	LTCF	2009 (6m)	All cases	Lab+symp	Various	295	1.15 (1.03-1.29)	
Silva (2013)	Brazil	ICU	2005-09	All cases	Lab+symp	EIA	15	3.26 (1.96-5.40)	1.39 (0.84-2.31)
Souza Dias (2010)	Brazil	ICU	2002-03	Onset in ward	Lab+symp	EIA Tox A&B, Cul	51		7.90 (6.00-10.39)
Souza Dias (2010)	Brazil	ICU	2002-03	All cases	Lab+symp	Cul, PCR	55		8.52 (6.54-11.10)
Dodek (2013)	Canada	ICU	2006-11	Onset in ward	Lab+symp	EIA Tox, Cyt, PCR	236		15.41 (13.56-17.51)
Elligsen (2012)	Canada	ICU	2008-09	Nosocomial	No details	No details	59	11.85 (9.18-15.3)	6.79 (4.16-11.1)
Faires (2014)	Canada	ICU	2006-11	Onset in ward	Lab+symp	EIA Tox A&B, GDH	11	2.68 (1.48-4.84)	
Teltsch (2011)	Canada	ICU	2000-05	Onset in ward	Lab+symp		135	29.3 (24.75-34.69)	
Wang (2014)	China	ICU	2012-13	No details	Lab+symp	PCR	31	25.20 (17.72-35.83)	24.28 (17.07-34.52)
Barbut (2007)	France	ICU	2000-04	All cases	Lab+symp	Cyt, Cul, EIA, PCR	25	10.00 (6.76-14.80)	
Khanafer (2013)	France	ICU	2007-11	All cases	Lab+symp	Cyt, EIA	40		12.94 (9.49-17.64)
Zahar (2012)	France	ICU	1999-09	Onset in ward	Lab+symp	EIA	47	36.00 (27.05-47.91)	
Buendgens (2014)	Germany	ICU	1999-10	Onset in ward	Lab+symp	EIA and/or PCR Tox A/B	110		14.91 (12.38-17.97)
Weitzel-K (2008)	Germany	ICU	2007	Onset in ward	Lab+symp	Tox, Cul	160	7.52 (6.44-8.78)	
Weitzel-K (2008)	Germany	ICU	2007	All cases	Lab+symp	Tox, Cul	193	9.07 (7.88-10.44)	
Czepiel (2015)	Poland	ICU	2008-14	All cases	Lab+symp	Antigen, ELISA Tox A/B	72		24.4 (19.37-30.74)
Khan (2012)	Qatar	ICU‡	2006-09	No details	Lab+symp	EIA Tox A/B	*NR*	*0.09-0.3*	
Alvarez-Lerma (2014)	Spain	ICU	2012	Hosp	Lab+symp	GDH, EI ATox A/B, Cyt, PCR	31	2.06 (1.45-2.93)	1.59 (1.12-2.26)
Bouza (2015)	Spain	ICU	2003-1	All cases	Lab+symp	Tox Rapid Test	117	18.24 (15.22-21.86)	
Salva (2014)	Spain	ICU	2010-11	All cases	Lab+symp	EIA Tox A/B	7	3.10 (1.48-6.50)	3.62 (1.72-7.58)
Thibault (2013)	Switzerland	ICU		Onset in ward	Lab+symp	No details	2	9.81 (2.45-39.24)	4.74 (1.19-18.95)
Chung (2010)	Taiwan	ICU	2007-08	No details	Lab+symp	EIA Tox A/B	26	11.06 (7.35-16.64)	
Hsu (2006)	Taiwan	ICU	2003-04	No details	Lab+symp	EIA Tox A/B	6	10.12 (4.55-22.52)	
Lee (2012)	Taiwan	ICU	2009-10	All new cases	Lab+symp	GDH+EIA Tox A/B	4	7.92 (2.97-21.1)	
Ang (2008)	UK-Eng	ICU	2004-07	>48 hours ICU admission	Lab+symp	Cyt A&B	62		33.48 (26.10-42.94)
Shears (2010)	UK-Eng	ICU	2006-07	Onset in ward	Lab+symp	Cyt, EIA Tox A/B	11	24.00 (13.29-43.34)	
Wilson (2011) [a]	UK-England	ICU	2007	Onset in ward	Lab	No details	8	14.8 (7.4-29.6)	
Wilson (2011) [b]	UK-England	ICU	2007	Onset in ward	Lab	No details	8	6.2 (3.1-12.4)	
Reddy (2010)	UK-Scotland	ICU	2003-07	Hosp	Lab	Tox EIA	NR	6.23	
MoH Uruguay (2012)	Uruguay	ICU	2012	No details	S-Surv, Lab	No details	40	5.12 (3.76-6.98)	
Brown (2015)	US	ICU	2010-14	All cases	Lab+symp		62	9.52 (7.43-12.22)	
Lawrence (2007)	US	ICU	1997-99	>24h bef/onset in ICU	Lab+symp	Cyt, Tox A/B	40	32 (23.47-43.63)	15.2 (11.15-20.72)
McFarland (2007)	US	ICU	2004	No details	Lab+symp	EIA	NR	50.3	
Barletta(2014)	US	ICU	2001-08	Hosp	ICD9	No details	239		6.62 (5.83-7.51)
Nagaraja (2015)	US	ICU	2010-11	Onset in ward	Lab+symp	PCR Tox B gene	47	18.25 (13.71-24.29)	

Author	Country	Setting	Study Years	Parameters	Case ascertainment	Laboratory assay	Cases	CDI incidence rates and 95% confidence intervals	
								10,000 pt-days	1,000 admission
Passaretti (2013)	US	ICU	2007	Onset in ward	Lab+symp	No details	26	26.87 (18.29-39.47)	
Popovich (2009)	US	ICU	2004-05	Onset in ward	Lab	No details	6	8.9 (4-19.81)	
Sabbah (2015)	US	ICU	2003-08	Onset in ward	Lab	No details	347		34.66 (31.2-38.51)
Ahmetagic (2013)	Bosnia	Internal med	2009-12	Onset in ward	Lab+symp	ELISA Tox A/B	86	3.30 (2.63-4.14)	3.15 (2.51-3.96)
Faires (2014)	Canada	Internal Med	2006-11	Ward acquired (>48 hours)	Lab+symp	EIA Tox A/B	27	2.16 (1.48-3.15)	
Lee (2014)	Canada	Internal med	2012-13	>3 days/IP (pre-intervention)	Lab only	PCR	40	24.1 (17.7-32.9)	35.1 (25.8-47.9)
Balihar (2014)	Czech Rep	Internal med	2006-08	Onset in ward	Lab+symp	EIA	45	3.01 (2.24-4.03)	
Balihar (2014)	Czech Rep	Internal med	2006-08	All cases	Lab+symp	EIA	56	3.74 (2.88-4.86)	
Weitzel-K (2008)	Germany	Internal med	2007	Onset in ward	Lab+symp	Tox, Cul	1112	7.99 (7.53-8.47)	
Weitzel-K (2008)	Germany	Internal med	2007	All cases	Lab+symp	Tox, Cul	1687	12.12 (11.55-12.71)	
Kurti (2015)	Hungary	Dept. med	2010-13	All cases	Lab	Cyt, Cul	247	25.65 (22.64-29.06)	
Bishara (2013)	Israel	Internal Med	2011	All cases	Lab+symp	EIA Tox A/B	178		28.25 (24.4-32.72)
Nseir (2013)	Israel	Internal med	2011	Onset in ward	Lab+symp	EIA Tox	197		28.76 (25.01-33.07)
Mellace (2013)	Italy	Internal med	2008-09	Onset in ward	Lab+symp	EIA Tox, Cul, Cyt	146	23.3 (19.81-27.4)	24.06 (20.46-28.3)
Sansone (2009)	Italy	Internal med	2007-08	Onset in ward	Lab+symp	EIA	13	6.23 (3.62-10.73)	4.98 (2.89-8.58)
Garcia (2007)	Peru	ICU, Internal med	2005-06	All cases	Lab+symp	ELISA	55		12.90 (9.90-16.80)
Czepiel (2015)	Poland	Internal Med	2008-14	All cases	Lab+symp	ELISA Tox A/B	890		4.04 (3.78-4.31)
Khan (2012	Qatar	Internal med	2006-09	No details	Lab+symp	EIA Tox A/B	*NR*	*0.5*	
Koh (2007)	Singapore	Internal med	2002-03	All cases	Lab+symp	EIA ToxA/B, PCR, Cul	13		3.25 (1.88-5.59)
Marco-Martínez (2015)	Spain	Int Med	2005-10	All cses	ICD9		7247		2.16 (2.11-2.21)
Chung (2010)	Taiwan	Internal med	2007-08	All cases	Lab+symp	EIA Tox A/B	60	1.05 (0.82-1.35)	
Shears (2010)	UK-Eng	Internal med	2006-07	Onset in ward	Lab+symp	Cyt, EIA Tox A/B	43	11.00 (8.16-14.83)	
Dubberke (2009)	US	General med	2006-11	Onset in ward	Lab+symp	EIA Tox A/B	148	16.00 (13.62-18.80)	

Notes: LTCF: Long-term care facility, ICU: Intensive Care Unit, *Mean: 31.2 CDI cases per 10,000 patients-days but information could not be confirmed by author. ICD9: code 008.45, S-Surv: Sentinel surveillance, Tx: Treatment, NR: Not reported. Pawar (2012) and Campbell also report rates for initial and recurrent episodes: 3.24 cases per 10,000 patient-days (95CI 2.99-3.51) and 3.74 (95CI 2.19-6.38), respectively. ‡medical, surgical, trauma/coronary care. (i) incident cases only (i+r): incident and recurrent cases, IP: in-patient, ICD9: 008.45, EIA: Enzyme Immunoassay, Tox: Toxin, &: and, /: orCyt: Cytotoxin, GDH: glutamate dehydrogenase, Cul: Culture, PCR: polymerase chain reaction. NAAT: nucleic acid amplification test Lab: Laboratory confirmation, Symp: symptoms mentioned, including at least one of the following unformed or loose stool, diarrhoeal disease, endoscopic or histologic features, Tx: Treatment.

Supplementary Table 7. CDI incidence, all outcomes (children)

Reference	Country	Study years	Age (Y)	Case definition	Case ascertainment	Laboratory assay	CDI cases	CDI Incidence rate (95% Confidence Interval)		
								10,000 patient-days	1,000 admission	100,000 population
Ahmetagic (2013)	Bosnia	2009-12		HCF-CDI	Lab+symp	ELISA ToxA&B	2	0.16 (0.02-1.14)‡	0.09 (0.01-0.64)	
PHA (2014)	Canada	2007-12	Children	HCF-CDI	Lab+symp	PCR, PFGE	580	4.25 (3.92-4.61)	2.27 (2.10-2.47)	
Schwartz (2014)	Canada	2008-12	1-17	HCF-CDI	Lab	Cyt, PFGE	222	8.30 (7.28-9.47)		
Sathyendran (2014)	N Zealand	2011-12	<15	All IP cases	Lab	EIA+GDH/PCR	46	2.00 (1.50-2.67)		
Duleba (2013)	Poland	2010-12	<18	HCF-CDI (>48 hours/IP<4 wks)	Lab+symp	PCR	28		6.01 (4.15-8.71)	
Jen (2012)	UK-England	1997-10	2-19	HCF-CDI	ICD10+≥72LOS		1549	0.67 (0.64-0.7)	0.15 (0.14-0.16)	
Reddy (2010)	UK-Scotland	2003-07	0-20	All IP	Lab		94	4.5 (3.68-5.51)		
Tschudin-S (2013)	US	2003-12	1-21	HCF-CDI (>48 hours/IP<4 wks)	Lab+symp	GDH+CytA/B/PCR; PCR	144		2.50 (2.12-2.94)	
Yin (2013)	US	2002-10	Children	HAI	Lab	No details	72	4.27 (3.39-5.38)		
Nagaraja (2015)	USA	2010-11	Pediatrics	>72 hours	Lab	PCR	17	7.09 (4.41-11.41)		
Benson (2007)	US	2001-06^	Children	All cases	Lab+symp	EIA Tox A/B	21	8.5 (5.56-13.07)		
Tartof (2014)	USA	2011-12	2-18	All cases	Lab	PCR	94	18.52 (15.13-22.67)		
Mitchell (2014)	Australia	2007-10	2-19	HO HCF-CDI (>48 hours)	Lab+symp	ELISA ToxA&B+PCR	9		1.80 (0.94-3.46)	
Sathyendran (2014)	N Zealand	2011-12	<15	HO	Lab	EIA+GDH/PCR	29	1.26 (0.88-1.81)		
Campbell (2009)	US	2006	Pediatric	HO HCF-CDI	Lab+symp	EIA, Cyt, GDH, a/o Cul	NR	*2.6*		
Tschudin-S (2013)	US	2003-12	1-21	HO HCF-CDI (>48 hours)	Lab+symp	GDH+CytA/B/PCR; PCR	103		1.78 (1.47-2.16)	
Duleba (2013)	Poland	2010-12	<18	CA-CDI (no IP 12wks)	Lab+symp	PCR	33		7.09 (5.04-9.97)	
Soes (2014)	Denmark	2009-11	2-19	CA-CDI	Lab+symp GP	Cul	6			5.42 (2.44-12.07)
Jen (2012)	UK-England	1997-10	2-19	CA-CDI	ICD10 no IP<90d		337		0.03 (0.027-0.03)	
CDC (2008)	US	2006	0-14	CA-CDI	Lab+symp	EIA Tox	18			2.71 (1.71-4.30)
Khanna (2013)	US	1991-09	<18	CA-CDI	ICD-9+Lab+symp	EIA, PCR + chart review	69			10.30 (8.14-13.04)
Lessa (2015)	US	2011	1-17	CA-CDI	Lab+symp	Tox,PCR	12,500			17.90 (14.10-21.40)
Tschudin-S (2013)	US	2003-12	1-21	CA-CDI	Lab+symp	GDH+CytA/B/PCR; PCR	38		0.66 (0.48-0.90)	
Lessa (2014)	USA	2010	1-17	CA-CDI	No IP <12 wks	Tox, PCR				13.6 (12-15.2)
Mitchell (2012)	Australia	2010-11	2-19	All cases IP	Lab+symp	See above	15			5.66 (3.41-9.39)
Kotila (2011)	Finland	2008	1-14	All cases IP	Lab+symp	Cul, Tox	137			16.40 (13.87-19.39)
Vesteinsdottir (2012)	Iceland	2010-11	0-17	All cases IP	Lab	ELISA Tox A&B	18			22.00 (13.86-34.92)
Duleba (2013)	Poland	2010-12	<18	All cases IP	Lab+symp	PCR	64		13.75 (10.76-17.56)	
Rodriguez-Pardo (2013)	Spain	2009	<15	All cases IP	Lab+symp	Tox A&B, Cul				*8.1*
Santiago (2015)	Spain	2010-11	<15	All cases IP	Lab+symp	Tox	19		3 (1.91-4.7)	
PHE	UK	2013-15	2-14	All cases IP	Lab+symp	Tox, PCR	278			3.44 (3.06-3.87)
Lessa (2014)	US	2010	1-17	All cases IP	Lab+symp	PCR				19.9 (17.2-22.6)
Tartof (2014)	US	2011-12	2-18	All cases IP	Lab	PCR	94	18.5 (15.13-22.67)	5.45 (4.35-6.82)	
Tschudin-S (2013)	US	2003-12	1-21	All cases IP	Lab+symp	GDH+CytA/B/PCR; PCR			3.50 (3.05-4.02)	
Argamany (2015)	US	2001-10	<18	All cases	ICD-9		825558		1.2 (1.2-1.2)	
Chen (2012)	US	2001-08	≥1*	Any position	ICD-9+Lab+symp	No details	231		4.30 (3.78-4.89)	
Khanna (2013)	US	1991-09	0-18	All cases	ICD-9+Lab+symp	EIA Tox + PCR	92			13.73 (11.20-16.85)
Kim (2008)	US	2001-06	<18	All cases	ICD-9+Lab+symp	EIA Tox	34141		3.30 (3.21-3.39)	
Lee (2012)	US	2000-08	<18	First 2 diagnoses	ICD-9	No details	*3665*		*0.57 (0.56-0.57)*	
Lucado (2012)	US	1993-09	<18	Any position	ICD-9	No details	8418		1.3 (1.27-1.33)	11.29 (11.05-11.54)
McDonald (2006)	US	1996-03	<15	Any position	ICD-9	No details	NR			9 (5-9)
Rhee (2014)	US	2010-12	1-17	All cases	Lab+symp	EIA Tox + GDH	197			41.74 (36.30-47.99)
Sammons (2013)	US	2006-11	1-18	All casses	ICD-9+Lab test	No details	5107		7.31 (7.11-7.52)	
Shaklee (2011)	US	2007 (6m)	<18	Any position	ICD-9	No details	119		4.37 (3.65-5.23)	
Shaklee (2011)	US	2007 (6m)	<18	All cases	ICD-9+Lab test	Tox, Cul	109		4.00 (3.32-4.83)	
Smith (2011)	US	2001-10	0-14	Any position	ICD-9	No details	690			7.13 (6.62-7.68)
VerLee (2012)	US	2002-08	<20	Any position	ICD-9	No details	170			1.12 (0.75-1.67)
Zilberberg (2010)	US	1997-06	0-17	Any position	ICD-9	No details	24037		0.93 (0.92-0.94)	
Zilberberg (2010)	US	2006	0-17	Any position	ICD-9	No details	98		1.45 (1.19-1.76)	

Notes: ^2 years data (2001: 5 cases, IR 10.24; 2006: 16, IR: 6.8 cases per 10,000 patient-days); ‡ Weighted mean, * <1 included if treated with metronidazole. Surv: Surveillance, HAI: Healthcare associated infection. AL: America, Latin, AN: America, North, EU: Europe, WP: Western Pacific, EM: Eastern Mediterranean, AF: Africa. (i) incident cases only (i+r): incident and recurrent cases, IP: in-patient, ICD9: 008.45, EIA: Enzyme Immunoassay, Tox: Toxin, &: and, /: orCyt: Cytotoxin, GDH: glutamate dehydrogenase, Cul: Culture, PCR: polymerase chain reaction. Lab: Laboratory confirmation, Symp: symptoms confirmation, including at least one of the following diarrhoeal disease, endoscopic or histologic features

Supplementary Table 8. CDI incidence, all outcomes (elderly)

Author	Country	Study years	Age (Years)	CDI category	Case ascertainment	Laboratory assay	CDI Cases	CDI incidence rates and 95% confidence interval		
								10,000 patient-days	1,000 admission	100,000 population
Koh (2007)	Singapore	2002-03 (5m)	≥60	HCF-CDI	Lab+symp	EIA Tox A/B, Cul	53		4.73 (3.61-6.19)	
PHE	UK England	2004-07	≥65	All IP cases	Lab	No details	202492	21.73 (21.64-21.83)		
HPS	UK Scotland	2006-14	≥65	All IP cases	Lab	No details	24841	7.67 (7.57-7.77)		
Jen (2012)	UK-England	1997-10	≥60	HCF-CDI	ICD9 + >72LOS	No details	173843	5 (4.98-5.03)	5.75 (5.72-5.78)	
HSC	UK-N.Ireland	2011	≥65	All IP cases	Lab	No details	1953	3.75 (3.59-3.92)		
Murphy (2012)	US	2000-2007	>75	HCF-CDI	ICD9 + Post Discharge history	No details	3379		4.22 (4.08-4.37)	
Tartof (2014)	US	2011-12	>65	All IP cases	Lab	PCR	3214	46.8 (45.2-48.5)	27.67 (26.66-28.72)	
Zilberberg (2011)	US	2007-08	>65	HCF-CDI	Lab (LabID database)	EIA Tox	4328	11.76 (11.4-12.1)	5.97 (5.79-6.15)	
Mitchell (2014)	Australia	2007-10	≥65	HO HCF-CDI	Lab+symp	EIA ToxA&/B, PCR, Cul	104		3.93 (3.24-4.76)	
Brown (2015)	US	2010-14	>60	HO HCF-CDI	Lab+symp	PCR	196	6.39 (5.56-7.35)		
Murphy (2012)	US	2000-2007	>75	HO HCF-CDI	ICD9 (First position+no PD)	No details	1352		1.69 (1.60.1.78)	
Zilberberg (2011)	US	2007-08	>65	HO HCF-CDI	Lab	EIA Tox	3114	8.46 (8.17-8.77)	4.29 (4.14-4.45)	
Mitchell (2012)	Australia	2010-11	>60	All cases	Lab+symp	EIA, PCR, Cul	161			142.76 (122.33-166.6)
Kotila (2012)	Finland	2008	≥65	All cases	Lab	Cul, Tox	4217			481.5 (442.8-520.1)
Lyytikainen -82009)	Finland	1996-04	>64	All cases	ICD10		582			108 (99.54-117.18)
Vesteinsdottir (2012)	Iceland	2010-11	≥65	All cases	Lab	ELISA Tox A&B	91			166 (135-204)
Rodriguez-Pardo (2013)	Spain	2009	>65	All cases	Lab+symp	Tox A&B, Cul	NR			67
PHE	UK-England	2013-15	≥65	All cases			21780			246.69 (243.52-249.9)
Kuntz (2012)	US	2007	>60	Any position	Lab+ ICD	EIA Tox	485			318.32 (291.22-347.95)
Lessa (2014)	US	2010	≥65	All cases	Lab+symp	Tox, PCR				631.8 (613.7-659.8)
Lessa (2015)	US	2011	≥65	All cases	Lab+symp	Tox, PCR	259800			627.7 (566.8-687.3)
Lucado (2012)	US	2009	≥65	Any position	ICD9	No details	219159			484.85 (482.83-486.89)
Mah (2011)	US	2007-2008	>59	All cases	Lab+symp	EIA Tox	144		7.64 (6.49-9.00)	
McDonald (2006)	US	1996-03	≥65	Any position	ICD9	No details	NR			228 (200-256)
Murphy (2012)	US	2000-2007	>75	All cases	ICD9	No details	7807		9.76 (9.55-9.98)	
Rogers (2014)	US	1991-07	>50	Any position	ICD9	No details	404			220.6 (193.3-248.8)
Smith (2011)	US	1999-08	≥65	Any position	ICD9	No details	11916			233 (229-238)
VerLee (2012)	US	2002-08	≥65	Any position	ICD9	No details	68686		68.5 (64.02-73.2)	7.35 (7.29-7.41)
Zilberberg (2008)	US	2000-05	≥65	Any position	ICD9	No details	617130			386.62 (384.7-388.55)
Zilberberg (2011)	US	2007-08	>65	All cases	Lab+symp	EIA Tox	5667		7.81 (7.61-8.01)	
Argamany (2015)	USA	2001-10	≥65	Any position	ICD-9		1538933		11.6 (11.58-11.62)	
Chopra (2015)	USA	2012	≥60	Any position	ICD-9		364		16.37 (14.77-18.14)	
Collins (2014)	US	2009-2011	≥65	CA-CDI	ICD9 First code+no IP>90 days)	No details	1566		1.81 (1.72-1.90)	
Murphy (2012)	US	2000-2007	>74	CA-CDI	ICD9 POA + (no IP>12 wks)	No details	4090		5.11 (4.96-5.27)	
Zilberberg (2011)	US	2007-08	>65	CA-CDI	Lab+symp (no IP>12 wks)	EIA Tox	1349		1.86 (1.76-1.96)	
Jen (2012)	UK-England	1997-10	≥60	CA-CDI	ICD9 + no IP <90days		17118		0.57 (0.56-0.58)	
Dial (2008)	Canada	1996-04	≥65	CA-CDI	ICD9 + IP history	No details	836			35 (32.71-37.45)
Soes (2014)	Denmark	2009-11	≥60	CA-CDI	Lab+symp (no IP>12 wks, <48h IP)	Cul	51			46.10 (35.03-60.66)
Marwick (2013)	UK	2008-09	≥65	CA-CDI	Lab+symp (i+r)	EIA Tox A&B	156			203.99 (174.36-238.65)
Taori (2014)	UK-Scotland	2010-11	≥65	CA-CDI	Lab no IP history <12 weeks	EIA ToxA/B, GDH	26			25.8 (17.56-37.9)
Kutty (2010)	US	2005	≥65	CA-CDI	No IP>8 wks, OP/<3-4 days IP	EIA Tox A&B				*146.4*
Lessa (2015)	US	2011	≥65	CA-CDI	Lab+symp (no IP>12 wks, <24h IP/OP)	Tox, PCR	60500			146.2 (124.0-167.2)
Lessa (2014)	USA	2010	≥65	CA-CDI	Lab+symp (no IP>12 wks, <24h IP/OP)					114.4 (104.1-124.7)

Notes: EIP: Emerging infections Program (Centers for Disease Control), HCUP: Healthcare Cost and Utilization Project, ICD10: A047, ICD9: 008.45, PD: Post-discharge, IP: In-patient, NR: not reported, *Daneman by decades (61-70, 71-80, 81-90), Surv: Surveillance, (i) incident cases only (i+r): incident and recurrent cases, IP: in-patient, ICD9: 008.45, EIA: Enzyme Immunoassay, Tox: Toxin, &: and, /: orCyt: Cytotoxin, GDH: glutamate dehydrogenase, Cul: Culture, PCR: polymerase chain reaction. Lab: Laboratory confirmation, Symp: symptoms confirmation, including at least one of the following diarrhoeal disease, endoscopic or histologic features. POA: Present on admission

Supplementary Document 9. CDI density incidence (per 10,000 patient-days) from multicentre European studies

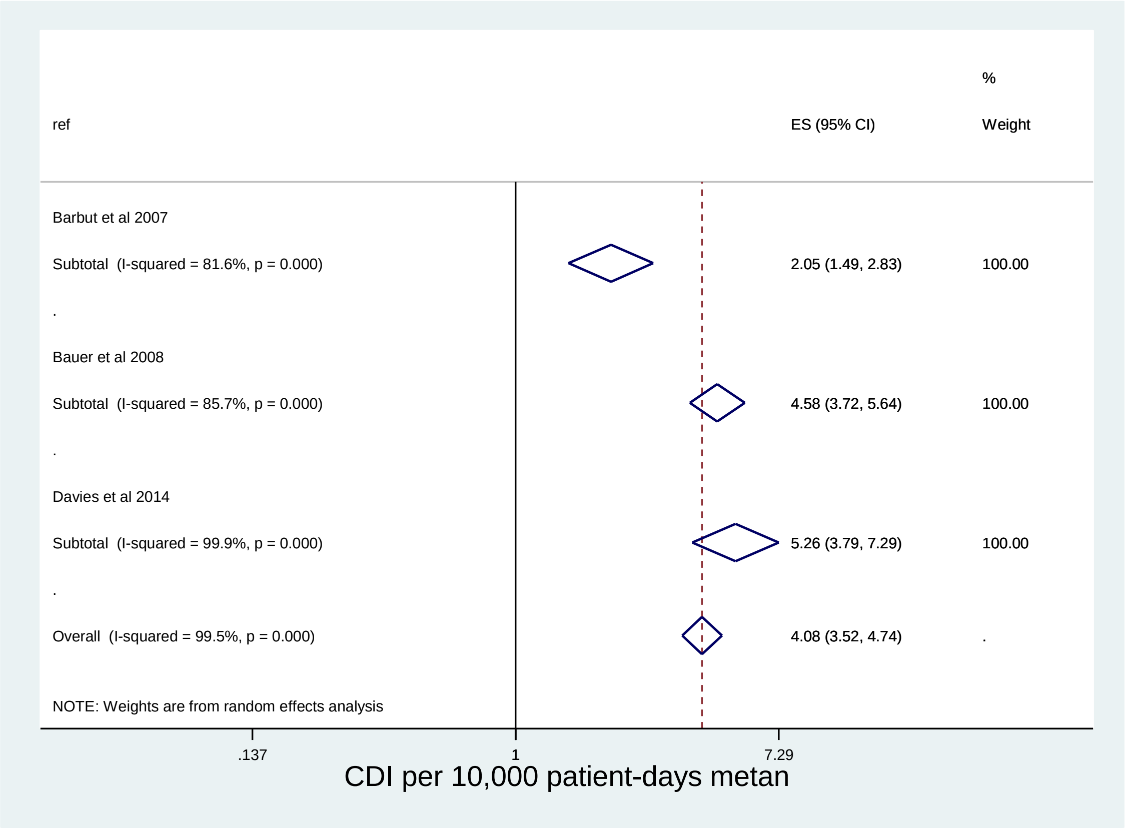

Supplement: Online Supplementary Document [file jogh-09-010407-s001.pdf]
